# Evolvability and trait function predict phenotypic divergence of plant populations

**DOI:** 10.1073/pnas.2203228120

**Published:** 2022-12-29

**Authors:** Øystein H. Opedal, W. Scott Armbruster, Thomas F. Hansen, Agnes Holstad, Christophe Pélabon, Stefan Andersson, Diane R. Campbell, Christina M. Caruso, Lynda F. Delph, Christopher G. Eckert, Åsa Lankinen, Greg M. Walter, Jon Ågren, Geir H. Bolstad

**Affiliations:** ^a^Department of Biology, Biodiversity Unit, Lund University, Lund 223 62, Sweden; ^b^School of Biological Sciences, University of Portsmouth, Portsmouth PO1 2DY, UK; ^c^Institute of Arctic Biology, University of Alaska, Fairbanks, AK 99775; ^d^Department of Biology, Centre for Ecological and Evolutionary Synthesis, University of Oslo, Oslo 0316, Norway; ^e^Department of Biology, Centre for Biodiversity Dynamics, Norwegian University of Science and Technology, Trondheim 7491, Norway; ^f^Department of Ecology and Evolutionary Biology, University of California, Irvine, CA 92697; ^g^Department of Integrative Biology, University of Guelph, Guelph, ON N1G 2W1, Canada; ^h^Department of Biology, Indiana University, Bloomington, IN 47405; ^i^Department of Biology, Queen’s University, Kingston, ON K7L 3N6, Canada; ^j^Department of Plant Protection Biology, Swedish University of Agricultural Sciences, 234 22 Lomma, Sweden; ^k^School of Biological Sciences, Monash University, Melbourne 3800, Australia; ^l^Department of Ecology and Genetics, Evolutionary Biology Centre, Uppsala University, 753 26 Uppsala, Sweden; ^m^Norwegian Institute for Nature Research, Trondheim 7485, Norway

**Keywords:** adaptation, evolvability, genetic constraints, macroevolution

## Abstract

Understanding the causes and limits of population divergence in phenotypic traits is a fundamental aim of evolutionary biology, with the potential to yield predictions of adaptation to environmental change. Reciprocal transplant experiments and the evaluation of optimality models suggest that local adaptation is common but not universal, and some studies suggest that trait divergence is highly constrained by genetic variances and covariances of complex phenotypes. We analyze a large database of population divergence in plants and evaluate whether evolutionary divergence scales positively with standing genetic variation within populations (evolvability), as expected if genetic constraints are evolutionarily important. We further evaluate differences in divergence and evolvability–divergence relationships between reproductive and vegetative traits and between selfing, mixed-mating, and outcrossing species, as these factors are expected to influence both patterns of selection and evolutionary potentials. Evolutionary divergence scaled positively with evolvability. Furthermore, trait divergence was greater for vegetative traits than for floral (reproductive) traits, but largely independent of the mating system. Jointly, these factors explained ~40% of the variance in evolutionary divergence. The consistency of the evolvability–divergence relationships across diverse species suggests substantial predictability of trait divergence. The results are also consistent with genetic constraints playing a role in evolutionary divergence.

## Significance

Variation in phenotypic traits among conspecific populations is common in nature and often, but not always, reflects adaptation to the local environment. Although natural selection is a potent force driving local adaptation, the genetic architecture of complex phenotypes can both slow down and deflect the approach of populations to their local adaptive optima. We compiled and analyzed a large database of population divergence in phenotypic traits paired with estimates of standing genetic variation (evolvability) within populations to reveal consistent positive relationships between evolutionary divergence and evolvability. When combined with data on trait function, evolvability estimates explain ~40% of the variation in population divergence. These results demonstrate substantial predictability of trait divergence and are consistent with genetic constraints on evolutionary divergence.

Standing genetic variation within populations is the fuel for evolutionary response to natural selection, and hence the divergence of populations and species. Studies of trait divergence among populations thus provide opportunities for understanding the link between microevolution observed within populations and macroevolution observed at the level of populations and species. There is ample evidence that adaptation can occur rapidly when environments change (1–4) and that local adaptation is common, although not universal (5–9). These observations suggest that trait divergence can be understood by knowing which environmental factors affect the phenotypic optimum for a given trait and the extent of variation in these environmental factors among populations. This narrative implicitly assumes that most phenotypic traits are sufficiently evolvable, and consequently no limit to trait divergence due to genetic constraints. Understanding the potential role of constraints in evolution thus requires studies of standing genetic variation within populations and how it relates to population divergence.

Most phenotypic traits exhibit substantial standing genetic variation, yet the amount of variation varies among traits (10, 11). Because traits rarely evolve independently, the divergence of any trait or combination of traits may also be constrained by genetic correlations with traits under conflicting selection (12–19). The genetic-constraint hypothesis posits that the genetic architecture of complex phenotypes channels phenotypic evolution along directions of high genetic variation in multivariate trait space, thus yielding a positive relationship between divergence and evolvability (13, 20–24). Most existing analyses are at least partly consistent with this expectation (e.g., refs. 22, 25–32), suggesting that evolvability provides a reliable predictor of population divergence. If true, this means that studies of standing variation within populations are both relevant and necessary for understanding macroevolutionary patterns such as population and species divergence.

Phenotypic traits involved in different functions (e.g., reproduction vs. physiological maintenance) often differ in both their evolvabilities and the patterns of selection acting on them (10, 33, 34). Consequently, a more predictive understanding of population divergence may arise from explicit consideration of trait function. For example, due to their central role in plant–pollinator interactions and plant reproduction, floral traits differ in important ways from other plant phenotypic traits. The selection for effective pollen transfer is expected to lead to limited phenotypic variation in floral traits within populations (35), especially in those traits directly involved with the fit of flowers to pollinators (10, 36–39). Floral traits also tend to have less additive genetic variance than vegetative traits and thus lower evolutionary potential (10). The divergence of floral phenotypes among populations likely depends on the extent to which pollinator communities vary geographically, at least for those traits directly involved in pollen transfer (40–42). In contrast, population divergence in vegetative traits and floral traits not directly involved in pollen transfer may depend more on variation in the abiotic environment (43, 44), herbivory or plant–plant interactions (45). Differences in divergence observed under natural field conditions may also result from differences in plasticity (9, 46), because vegetative traits generally exhibit higher plasticity than do floral traits (36–39). Furthermore, more evolvable traits tend also to be more plastic (47–49), which could exaggerate evolvability–divergence relationships across trait types. Comparing patterns of divergence among trait categories may thus yield insights into the relative importance of natural selection, evolvability, and plasticity in evolutionary divergence.

Population divergence in plants may also depend on the reproductive systems of the diverging populations (50). Because selfing species rely less on pollinators for seed production, they may experience relaxed selection for advertisements and rewards involved in attracting pollinators, and different populations of a selfing species might experience similar patterns of selection, such as selection for reduced herkogamy (anther–stigma separation), reward production and advertisements, as seen in the “selfing syndrome” (51). This could lead to convergent adaptive optima, and thus reduced divergence compared to outcrossing species. On the other hand, we do not necessarily expect covariation between mating systems and selection on vegetative traits, although the tendency for selfing to occur in stressful and frequently disturbed environments may cause an association between traits related to mating systems and stress tolerance (e.g., rapid reproduction; 52). Compared to outcrossing species, selfers are also expected to have less genetic variation (53–55) and, hence, lower evolvability and lower potential for adaptation (the “dead-end” hypothesis, refs. 56 and 57–58). The evidence for limited evolvability in selfing species is weak (53, 59–62), however, and the influence of mating systems on patterns of divergence remains an empirical question.

Here, we assess the roles of trait function, mating system, and evolvability as predictors of phenotypic divergence of plant populations. To this end, we compiled paired data on within- and among-population variances in plant traits, classified traits into functional categories (vegetative vs. reproductive), and species into mating-system categories (selfing vs. mixed-mating vs. outcrossing). First, we compared patterns of population divergence among trait functional groups and among species differing in mating systems. Second, we performed univariate and multivariate meta-analyses of the relationship between evolutionary divergence and evolvability (within-population genetic variance) and compared the resulting patterns among trait and mating-system categories. Comparing the univariate and multivariate analyses allows us to assess whether genetic correlations obscure evolvability–divergence relationships in univariate analyses. We estimated the slope of evolvability–divergence relationships, which allowed us to link the results to specific predictions of distinct macroevolutionary models (e.g., neutral vs. optimum-tracking models; see refs. 20 and 22, and *Discussion*). Third, to assess the potential influence of phenotypic plasticity on the observed relationship between divergence and evolvability, we asked whether evolvability–divergence relationships change when population divergence is assessed in natural populations vs. in an outdoor common garden or in a greenhouse. Finally, to complement the trait-focused analyses, we asked whether populations tend to diverge along multivariate phenotypic directions associated with above-average evolvability, as is also expected if genetic constraints are evolutionarily important.

## Results

### Patterns of Population Divergence.

**Fig. 1.**
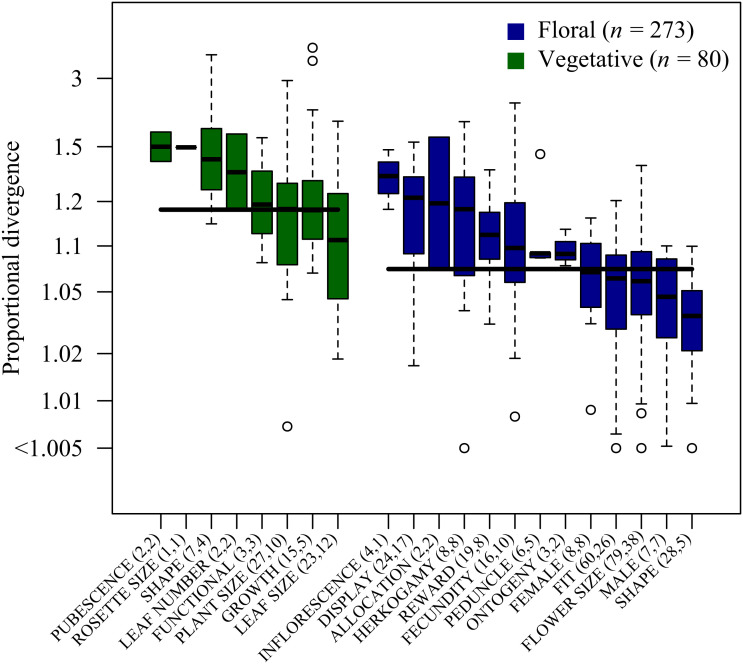
Proportional population divergence of vegetative (green boxes) and floral (blue boxes) traits. The *y*-axis gives the proportional divergence of an average population, where a value of 1.1 indicates that the trait mean of an average population has evolved to be c. 10% larger or smaller than the grand mean. Thick lines across boxes indicate the median of each trait category, and thick lines within boxes indicate median values for each trait subcategory. Boxes extend from the first to third quartile, range bars extend to 1.5 times the interquartile range, and data points outside this range are shown as open circles. Sample sizes are given in parentheses for each trait subcategory, with the first number giving the number of estimates, and the second number the number of unique studies. Trait categories are defined in *SI Appendix*, Tables S1 and S2.

To quantify and compare patterns of population divergence, we defined a “divergence study” as a set of population means measured as part of the same field survey or common-garden experiment. In total, we analyzed 48 such divergence studies comprising 2666 trait means from 314 populations of 33 species representing 20 families (*SI Appendix*, Appendix 1). We quantified the magnitude of divergence in each trait on a proportional scale by the factor *d_P_*, where the trait mean of the average population has evolved to be $d_{P}$ times larger or $1/d_{P}$ times smaller than the grand mean across populations. Across all divergence studies, the 273 floral traits had diverged by a median factor of *d_P_* = 1.070 ± 0.005 SE (i.e., evolved by approximately 7% in magnitude) and the 80 vegetative traits had diverged by a median factor of 1.176 ± 0.018 (Fig. 1). The greater divergence of vegetative traits than of floral traits was well supported statistically (ΔAIC = 5.21) and held when restricting the analysis to linear size measures only (*d_P_* = 1.069 ± 0.006 for floral traits vs. 1.137 ± 0.023 for vegetative traits, ΔAIC = 15.91).

Median divergence was largely independent of mating system, with only a weak tendency for greater divergence for selfing species (*d_P_* = 1.098 ± 0.010, *n* = 77) than for mixed-mating (*d_P_* = 1.083 ± 0.004, *n* = 186) and outcrossing species (*d_P_* = 1.072 ± 0.014, *n* = 102). Median divergence was similar whether assessed in the greenhouse (*d_P_* = 1.089 ± 0.004, *n* = 245) or in the field (*d_P_* = 1.081 ± 0.010, *n* = 93).

### Univariate Evolvability–Divergence Relationships.

In our meta-analysis of 38 divergence studies of 26 species, mean-scaled evolvability emerged as the principal predictor of evolutionary divergence (Table 1). Overall, divergence increased by 9.8% for a 10% increase in evolvability (attenuation-corrected slope on log-log scale = 0.98 ± 0.13). The slopes for individual divergence studies varied around this mean with an average deviation of 0.60, as quantified by the random-slope SD (Table 1). Observed divergence increased by 2.0% for a 10% increase in the geographic distance separating populations (slope on log-log scale = 0.20 ± 0.09), and by 4.4% for a 10% increase in the number of populations studied (slope on log-log scale = 0.44 ± 0.25).

**Table 1.** Parameter estimates ± SE from models describing population divergence (*d* = the variance of ln-transformed population means) as a function of evolvability (*e*), the number of populations (npop), and the maximum geographic distance between populations (dist)

Model: Subset	Fixed effects							
	Intercept	log (*e*)	log (npop)	log (dist)	Random-slope SD	ΔAIC	*r*^2^_M_ (%)	*r*^2^_C_ (%)
Baseline								
Overall	−5.11 ± 0.26	0.76 ± 0.10	0.44 ± 0.25	0.20 ± 0.09	0.60	0.00	37.3	87.1
Trait groups								
Floral traits	−5.21 ± 0.27	0.78 ± 0.11	0.56 ± 0.22	0.26 ± 0.09	0.60	1.39	39.6	89.0
Vegetative traits	−5.49 ± 0.30	0.91 ± 0.13						
Mating systems														
Selfing species	−4.91 ± 0.55	0.66 ± 0.26							0.43 ± 0.26	0.19 ± 0.10	0.62	6.34		
Mixed-mating species	−5.48 ± 0.44	0.73 ± 0.16											38.1	87.9
Outcrossing species	−4.92 ± 0.42	0.85 ± 0.17											
Study environments																
Field	−5.46 ± 0.61	1.16 ± 0.22	0.42 ± 0.28	0.15 ± 0.12	0.59								−1.11		
Common garden	−6.11 ± 0.75	0.87 ± 0.35				37.0								86.4
Greenhouse	−4.93 ± 0.28	0.62 ± 0.12											

The intercept gives the natural logarithm of the expected divergence when all covariates are at their mean value. A value of −5.11 (for the baseline model) translates into an expected proportional divergence of 1.064. The slope for log(*e*) reported here is not corrected for attenuation bias. The more complex models include the interaction between evolvability and the focal factor, and thus test for heterogeneity of slopes. The random-slope SD is for the slope of the evolvability–divergence relationship among divergence studies. The column ΔAIC gives the difference in AIC between the baseline model and the more complex models, with positive values indicating support for the baseline model over the more complex model.The *r*^2^_M_ gives the percent variance explained by the fixed effects, and *r*^2^_C_ gives the percent variance explained jointly by the fixed and random effects.

**Fig. 2.**
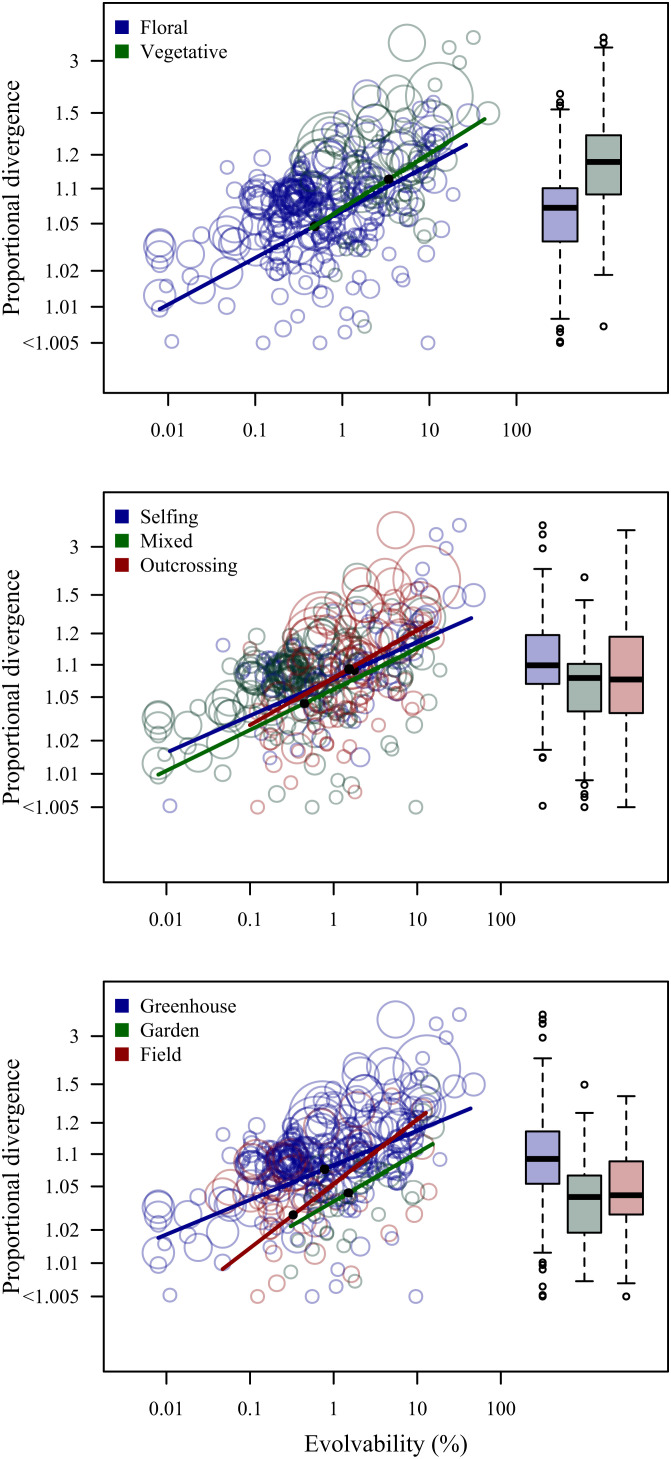
Univariate evolvability–divergence relationships for floral vs. vegetative traits, selfing vs. mixed-mating vs. outcrossing species, and populations measured in the greenhouse vs. outdoor common garden vs. field. Circle sizes are proportional to the square root of the number of populations studied. Regression lines show the estimated relationships, and solid dots indicate the expected divergence at the median evolvability in each group. See Table 1 for parameter estimates. The *y*-axis gives the proportional divergence of an average population from the grand mean (Fig. 1).

The slope of the evolvability–divergence relationship was steeper for vegetative traits than for floral traits (contrast = 0.13 ± 0.09; Fig. 2), but this difference was poorly supported statistically (Table 1). Similarly, the slope was not detectably dependent on mating systems or the environment in which divergence was assessed (Fig. 2 and Table 1), although slopes were steeper when divergence was assessed in the field (contrast from greenhouse = 0.53 ± 0.25) or outdoor common garden (contrast = 0.25 ± 0.37) than in the greenhouse.

### Multivariate Evolvability–Divergence Relationships.

We performed multivariate analyses for a set of 40 paired G- and D-matrices, where **G** is the mean-scaled within-population genetic variance matrix and **D** is the proportional (ln-scale) among-population variance matrix. We estimated the regression slope of divergence on evolvability on a log-log scale for a set of focal phenotypic directions (trait combinations) defined using four different approaches: 1) the original traits (equivalent to the univariate analyses), 2) the eigenvectors of **G**, 3) the eigenvectors of **D**, and when available, 4) the eigenvectors of the phenotypic variance matrix, **P**.

**Fig. 3.**
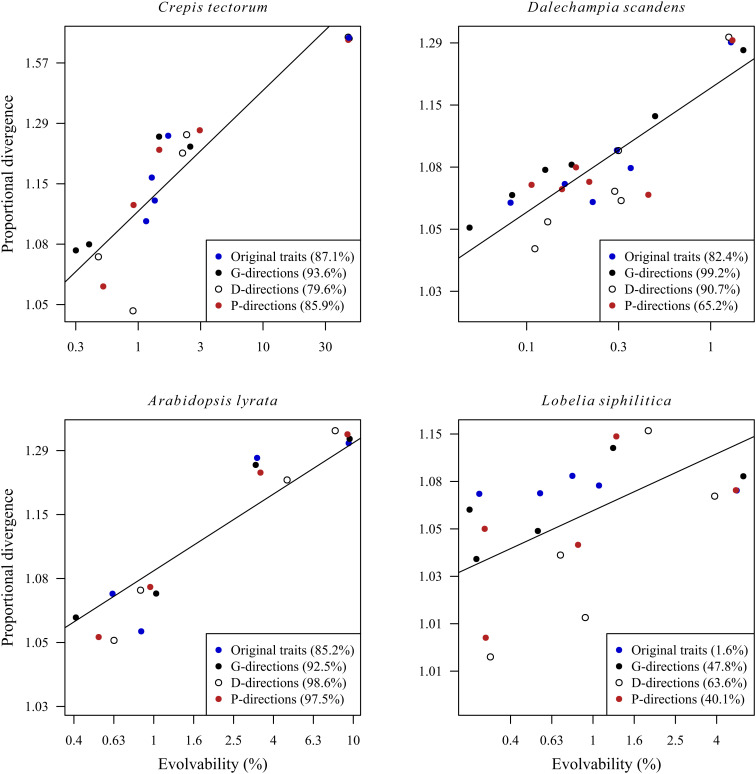
Examples of evolvability–divergence patterns in multivariate phenotype space. The panels explore patterns of divergence for leaf traits among 51 *C. tectorum* populations, blossom traits among 16 Costa Rican populations of *D. scandens* s.l., flower-size and floral display traits among 10 populations of *L. siphilitica*, and floral and vegetative traits among four Scandinavian populations of *Arabidopsis lyrata*. Lines indicate a 1:1 evolvability–divergence relationship passing through the mean of the data points, illustrating the near-isometric scaling of divergence with evolvability in all four cases. The *y*-axis gives the proportional divergence of an average population from the grand mean (Fig. 1). Focal directions (“traits”) are defined along the diagonal of the matrices (i.e., the original trait measurements), along the eigenvectors of the G-matrix, along the eigenvectors of the D-matrix, and along the eigenvectors of the mean within-population P-matrix. Coefficients of determination (*r*^2^) for the evolvability–divergence relationships are given in parentheses.

In most cases, as illustrated by leaf-trait evolution in *Crepis tectorum* and floral-trait evolution in *Dalechampia scandens* (Fig. 3), population divergence scaled positively with evolvability regardless of the focal phenotypic directions (original traits, eigenvectors of **G**, **D** and **P**), indicating overall similarity between the G- and D-matrices. In other cases, as illustrated by floral-trait evolution in *Lobelia siphilitica* (Fig. 3), the regression of divergence on evolvability was nearly flat for the original traits, but positive when we projected the original traits onto the eigenvectors of the G**-**, D-, or P-matrix.

Consistent with the univariate analyses, positive evolvability–divergence relationships in studies including several trait functional classes were explained in part by correlated differences in both evolvability and divergence across trait categories. For example, Scandinavian *Arabidopsis lyrata* populations (Fig. 3) had diverged primarily in a pair of phenotypic directions representing overall plant size and flower display (linear rosette size and number of flowers), while less divergence had occurred in directions representing flower morphology. Heterogeneity in trait categories or dimensionality (e.g., combining linear measurements with areas, volumes, or counts) could not explain the overall patterns, however, because the median slope for studies including only comparable linear size traits fell close to the overall median (see *SI Appendix*, Appendix 2 for an extended analysis).

**Fig. 4.**
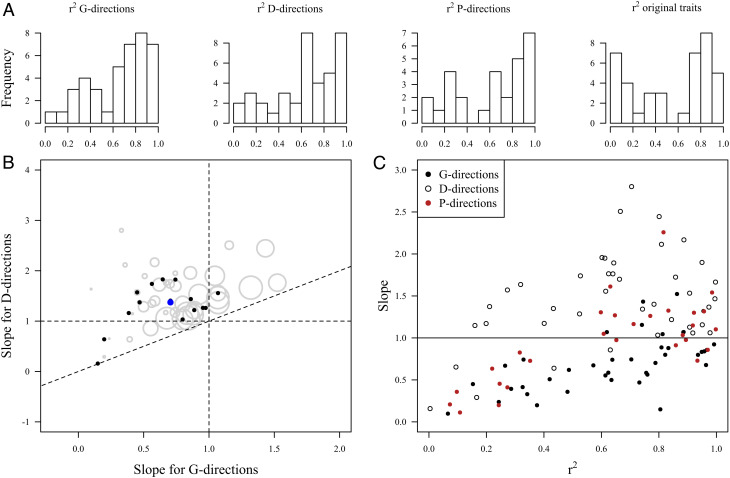
Summary of evolvability–divergence relationships (log-log regression slopes of population divergence on evolvability). The slopes are estimated for focal phenotypic directions represented by the original traits, and by the eigenvectors of the G-, D-, and P-matrices. (*A*) Frequency distribution of *r*^2^ values across individual cases. (*B*) Scatterplot of slopes estimated for the directions represented by the **G** and **D** eigenvectors. Gray circles are individual cases (paired G- and D-matrices), with circle size proportional to the overall *r*^2^ of the regression (including the original traits and the directions represented by the **G** and **D** eigenvectors). The black dots are species medians, and the blue dot is the median of the species medians. (*C*) Relationship between the slope of the evolvability–divergence relationships and the tightness of the relationship (*r*^2^) for each individual case.

When assessing all cases jointly (Fig. 4), three key patterns emerged. First, the predictive power of the evolvability–divergence relationships tended to be high, with *r*^2^ often greater than 50% for each set of focal directions. Second, as expected due to sampling variation in the estimated matrices (see *Methods*), the slopes of the evolvability–divergence relationships were steeper for the D-directions (error-weighed slope = 1.53 ± 0.10, median *r*^2^ = 0.65, *n* = 40) than for the G-directions (error-weighed slope = 0.70 ± 0.07, median *r*^2^ = 0.74, *n* = 40), and intermediate for the P-directions (error-weighed slope = 0.92 ± 0.12, median *r*^2^ = 0.68, *n* = 28). Third, the slopes for the G- and P-directions tended to converge on isometry (slope = 1) with increasing predictive power (*r*^2^) of the study.

Evolutionary divergence on longer time scales may be better predicted by considering the evolvability after accounting for genetic variation bound up in correlations with other traits, i.e., conditional evolvability. The relationships remained similar, however, when substituting conditional evolvability for evolvability (error-weighed slope = 1.61 ± 0.15 and 0.97 ± 0.14 for the D- and P-directions, respectively, see *SI Appendix*, Appendix 3 for an extended analysis).

Consistent with the univariate analyses, the multivariate evolvability–divergence relationships were broadly similar across trait categories, mating systems, and study environments (*SI Appendix*, Appendix 4).

### Evolvability along Observed Divergence Vectors.

**Fig. 5.**
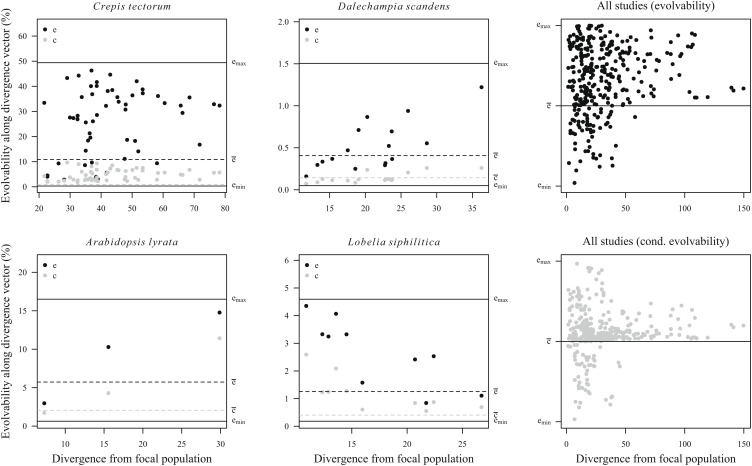
Patterns of evolvability (*e*) and conditional evolvability (*c*) along divergence vectors. Most populations have diverged in directions of above-average evolvability, and more so for populations that have diverged farther from the focal reference population in which a G-matrix was estimated. Horizontal lines give the mean ($\overset{-}{e}$), minimum ($e_{\min}$), maximum ($e_{\max}$) and mean conditional ($\overset{-}{c}$) evolvability of the focal-population G-matrix. The minimum and maximum evolvabilities are the evolvabilities along the leading and trailing eigenvectors. In the summary panels (*Right*), values are scaled proportionally between the mean and the minimum and between the mean and the maximum.

The analyses above focus on the evolvability–divergence relationship across traits within a G-matrix. If genetic constraints are important, we also expect populations to have diverged along phenotypic directions associated with above-average evolvability, so that the evolvability along the divergence vector between two populations is greater than the mean evolvability of the focal-population G-matrix. This was true for most of the example cases, with most populations diverging in directions of above-average evolvability, and often in directions of near-maximum evolvability (Fig. 5). The direction of maximum evolvability corresponds to the leading eigenvector of the G-matrix (*SI Appendix*, Appendix 5). Across all study systems, 80.9% of the populations had diverged in directions of above-average evolvability, and 85.2% in directions of above-average conditional evolvability (Fig. 5). The evolvability and conditional evolvability in the direction of divergence were, on average, 1.62 and 2.43 times the mean evolvabilities and conditional evolvabilities of the focal-population G-matrix (error-weighed log ratio = 0.48 ± 0.06 and 0.89 ± 0.11, respectively).

These analyses also show that populations that have evolved along directions of greater evolvability have evolved farther from the focal population. This was also true for most of the example cases, as indicated by the tendency for greater evolvability along the divergence vector with increasing divergence (Fig. 5). In contrast, *Lobelia* populations tended to have diverged along directions of high evolvability only when divergence was limited.

## Discussion

### Evolvability Predicts Phenotypic Divergence of Plant Populations.

Our analyses revealed consistent positive relationships between proportional divergence among plant populations and standing genetic variation within populations (i.e., mean-scaled evolvability; Figs. 2–4). Furthermore, they show that plant populations tend to diverge along multivariate phenotypic directions associated with above-average evolvability, and in no case have populations diverged extensively along directions of very low evolvability (Fig. 5). The inferred relationships held when environmental differences were controlled in the greenhouse or outdoor common garden and were not dramatically blurred by plasticity when assessed in situ in the field. In fact, the average slope of the evolvability–divergence relationship was steeper for the field data, suggesting that patterns of plasticity tend to align with directions of high evolvability and divergence (47–49). Despite relating current rather than historical evolutionary potential to historical divergence, these results suggest substantial predictability of evolutionary divergence patterns, and that studies of standing variation within populations are highly relevant for understanding not only microevolution, but also macroevolution.

The slope (scaling exponent) of the evolvability–divergence relationship derives its relevance from macroevolutionary models (20, 22). Estimating these slopes is challenging, but our meta-analyses suggest that the slopes are usually close to unity. This is consistent with recent estimates in animal systems (22, 26, 27), with models of neutral evolution, and with an optimum-tracking model with weak selection where the optimum moves according to an Ornstein–Uhlenbeck process (20). Additional information is required to separate the two latter possibilities. Under a neutral model, divergence is predicted to be *d* = 2*V_m_t*, while equilibrium evolvability is predicted to be *e* = 2*V_m_N_e_,* where *V_m_* is the mutational variance, *t* is the number of generations and *N_e_* is the effective population size (63). Hence, neutral evolution predicts *d/e = t/N_e_*. In our data, the average *d/e* ratio was close to unity (*SI Appendix*, Appendix 4). For the observed divergence to be consistent with neutral evolution, divergence times must therefore have been roughly similar to the effective population sizes of the diverging populations. Such divergence times seem unreasonably low because many of the studied species often occur in small populations (e.g., refs. 64 and 65–67), and effective population sizes are known to be considerably lower than census population sizes (68). In these cases, neutral evolution seems an unlikely explanation for the observed evolvability–divergence relationships. We are therefore left with an Ornstein–Uhlenbeck model with weak stabilizing selection in which the populations track the optima with a lag. Such a model (with weak selection) is consistent with a divergence–evolvability slope between 0.5 and 1, while under strong selection we would not expect any relationship (20).

A positive relationship between divergence and evolvability is also consistent with a scenario where evolvability increases during evolutionary divergence. In the multivariate case, alignment between G- and D-matrices may reflect evolution of **G** to align with the topography of the adaptive landscape (e.g., refs. 27, 69–73). While the issue of **G** evolving during evolutionary divergence is beyond our focus here, analyses in several systems of multiple independently estimated G-matrices yielded rather similar evolvability–divergence relationships, suggesting at least broad-scale similarity among G-matrices (e.g., ref. 74). Further insights into the role of selection in shaping **G** and potentially yielding evolvability–divergence relationships would require data on multivariate selection combined with the kinds of data analyzed here (75). Another unresolved issue is the role of gene flow among diverging populations in generating evolvability–divergence relationships. Under certain scenarios, gene flow could lead to greater within-population variation for traits exhibiting greater divergence among populations, although this requires relatively high rates of gene flow compared to local recombination rates and strength of selection driving local adaptation (76).

Although phenotypic population divergence is a multivariate process, our univariate analyses successfully revealed the general relationship between population divergence and evolvability. This suggests that the univariate traits analyzed represent well the ranges of evolvability and divergence in the study species. Multivariate analyses are not redundant, however, because the greater resolution of these analyses is sometimes needed to reveal clear relationships. This phenomenon could arise when, as illustrated by *Lobelia* floral traits (Fig. 3), the original traits are rather homogeneous and related to overall size. In these cases, the multivariate approach allowed us to decompose the phenotypic variation into more heterogeneous size vs. shape components, and thus to reveal a positive relationship between divergence and evolvability.

Conditional evolvabilities are expected to become increasingly strong predictors of divergence under continued directional selection (77). In the present analyses, the evolvability–divergence relationships were rather similar for unconditional and conditional evolvabilities, and we failed to detect an increase in the predictive power of conditional evolvabilities using the maximum geographic distance among populations as a rough proxy of divergence time. Further testing of this hypothesis requires phylogenetic information allowing better estimates of the time of divergence of populations. Finally, we chose, for simplicity, to compute conditional evolvabilities with respect to the entire G-matrix, but it could also be informative to consider more nuanced scenarios of directional vs. stabilizing selection on specific traits (13).

### Phenotypic Divergence Is Related to Trait Function.

Although the evolvability–divergence relationships were broadly similar across trait functional groups, additional insights arose from considering trait function. Greater divergence of vegetative than floral traits (Fig. 1) could reflect greater evolvability (10), greater plasticity (e.g., ref. 46), or greater variation in adaptive optima for vegetative compared to floral traits (e.g., ref. 78). The selection on the physiological function of vegetative traits will typically be mediated by abiotic environmental factors (34), while the selection on floral traits functionally involved in pollination is generally thought to be mediated by pollinators (79, 80) or other biotic interactors (33, 81). Greater variation in optima for vegetative traits than for floral traits would thus be expected if abiotic environments vary more among populations than do biotic-interactor communities. Variation in abiotic environments could also lead to greater observed divergence of vegetative traits than of floral traits due to plasticity, because vegetative traits generally exhibit higher plasticity than floral traits (36–39). Most of the data on vegetative traits we considered were collected in greenhouses, and plasticity therefore seems unlikely to explain the observed difference.

A recent meta-analysis invoked variation in precipitation patterns as a strong driver of variation in selection, but did not consider in any detail whether this relationship differs among trait groups (82). While it seems reasonable that this association could reflect variation in selection on vegetative traits associated with physiological performance across different precipitation environments, a similar association for floral traits might well be influenced by the effects of precipitation patterns on communities of pollinators and other interactors (83). Furthermore, pollen limitation is a key driver of variation in the opportunity for selection on floral and other pollination traits (84) and may also vary along precipitation gradients (85). Finally, evolvability and adaptation are not mutually exclusive mechanisms. Further studies combining detailed data on pollinator communities and abiotic environments across populations (e.g., ref. 86) would be a good start at addressing the importance of variation in optima, especially when combined with the characterization of fitness functions across environments.

### Population Divergence Is Largely Independent of Mating System.

We detected only limited differences in divergence patterns and evolvability–divergence relationships between selfing, mixed-mating, and outcrossing species and, if anything, selfing species tended to have diverged more than mixed–mating and outcrossing species. At face value, this observation is inconsistent with the expectation that selfing species have reduced adaptive potential (58). Selfing species in our dataset did not exhibit reduced evolvability compared to outcrossing species, and the results are therefore not at odds with the constraint hypothesis. The tendency for greater divergence for selfers could reflect differences in effective population size and gene flow. Selfers tend to exhibit much stronger genetic structure than outcrossers, due to strong genetic drift in highly selfing populations and/or reduced gene flow associated with reduced cross-pollination (87–89). Because reduced gene flow among populations is expected to promote local adaptation (90), this predicts increased opportunity for adaptation in selfers.

The pattern of greater divergence of selfing species was clearer for floral traits than for vegetative traits (*SI Appendix*, Appendix 4). Floral traits involved in pollen transfer in outcrossing species may often be under fluctuating and net stabilizing selection acting via flower-pollinator fit (35–39). This could explain the limited divergence of floral traits in outcrossing species, at least if pollinator identities are fairly constant across populations leading to convergent adaptive optima for floral traits functionally involved in pollination. For those floral traits not directly involved in pollen transfer, and more generally for selfing species, the divergence of floral traits could relate, for example, to variation in physiological costs of flower production across variable abiotic environments (see ref. 91).

While some support for the selfing-as-dead-end hypothesis stems from phylogenetic studies detecting reduced diversification rates of some self-compatible lineages compared to self-incompatible relatives (e.g., refs. 92 and 93), it is unclear whether similar trends exist for lineages with continuous variation in outcrossing rates (94, 95). Our results add to several observations that seem inconsistent with reduced adaptability of selfing species. First, a meta-analysis comparing the geographic range size of selfing and outcrossing species found that selfers tend to have larger geographic ranges than more outcrossing species (96), perhaps as a consequence of reduced reliance on pollinators and thus greater reproductive assurance when colonizing new environments (97). Second, a meta-analysis of reciprocal-transplant experiments failed to detect a difference between selfing and outcrossing lineages in the degree of local adaptation (60).

### Evolvability, Trait Function, and the Predictability of Population Divergence.

The consistent positive relationship between historical evolutionary divergence and current evolvability suggests that evolvability estimates carry appreciable predictive power beyond a few generations, thus providing a tool for predicting future adaptation in the event of environmental change. The divergence patterns for floral and vegetative traits further underscore the importance of studies assessing relationships between phenotypic traits, relevant performance metrics such as pollination success for floral traits and nutrient acquisition or survival for vegetative traits, and fitness (80, 98). Finally, a positive relationship between divergence and evolvability is consistent with the importance of genetic constraints in evolutionary divergence. A critical question for further work is to what extent this pattern also reflects the evolution of evolvability during adaptive radiation, including the role of selection in shaping the structure of the G-matrix (75).

## Materials and Methods

### Data Collection.

We compiled data on variation in phenotypic traits within and among natural plant populations. We started by searching for available population-divergence data (population means and variances) for those species included in an updated version of a plant evolvability database compiled through systematic surveys of the literature (10, 59). To do so, we started from the original papers represented in the evolvability database, and subsequently tracked references to associated papers on the same system. We also added population-divergence data from our own study systems (*SI Appendix*, Appendix 1). All data are available at https://github.com/oysteiop/EvolDiv. The focal populations were measured in greenhouses, in outdoor common gardens, or as natural field populations. We included a few cases where populations were assigned to distinct ecotypes. We included traits measured on a true ratio scale (99) and excluded traits that could not be meaningfully subjected to mean-scaling (see below). This excluded many phenological traits measured on interval scales.

In the absence of molecular divergence data for most of the study systems, we estimated raw divergence rather than evolutionary rates. This assumes that populations within a study are of the same age, though we treated the maximum geographic distance among each set of populations as a rough proxy of divergence time. The maximum distance was computed from geographic coordinates when available, or otherwise approximated from maps in the original papers, or through correspondence with the authors.

To assess differences in divergence among trait groups, we classified traits as vegetative, floral (reproductive), and life-history related, and divided these further into sub-categories such as plant size vs. leaf size, and flower size vs. flower-pollinator-fit traits (*SI Appendix*, Appendix 1). To assess whether divergence depends on mating systems, we classified species as predominantly selfing (outcrossing rate *<* 0.2, or described by investigators as predominantly selfing), mixed mating (outcrossing rate between 0.2 and 0.8, or described as mixed mating), or predominantly outcrossing (self-incompatible, outcrossing rate *>* 0.8, or described as predominantly outcrossing).

### Quantifying Variation within and among Populations.

To compare within- and among-population variances, we expressed both on proportional scales. Mean-scaled genetic variance $I_{A}=V_{A}/{\bar{x}}^{2}$, where $V_{A}$ is the additive genetic variance and $\bar{x}$ is the trait mean is a useful measure of evolvability, because it corresponds to the predicted evolutionary response, in the percentage of the trait mean, to an episode of unit-strength selection (100). Similarly, we computed a measure of proportional divergence ($I_{D}$) as the variance of natural-log-transformed population means. These measures of evolvability and divergence extend conceptually to the multivariate case (13), and we will denote evolvability as *e* and divergence as *d*. To facilitate interpretation, we assumed a normal distribution for the log-transformed population means and expressed the expected proportional distance of a population from the grand mean as $d_{P}=exp\left(\sqrt{d}\sqrt{2\pi^{-1}}\right)$, where d is the estimated among-population variance. Multiplying $\sqrt{d}$ with $\sqrt{2\pi^{-1}}$ yields the expected value of a folded normal distribution, and taking the exponent returns the estimate to the arithmetic scale. On this scale, the trait mean of the average population is $d_{P}$ times larger or $1/d_{P}$ times smaller than the grand mean across populations. In other words, $d_{P}$ represents the magnitude of divergence on a proportional scale.

Expressing variances on a proportional scale corrects for different trait units, but proportional variances depend on trait dimensionality, so that areas, for example, tend to be more variable than linear measurements (101). We explore the sensitivity of our analyses to dimensionality in *SI Appendix*, Appendix 2.

### Evolvability, Conditional Evolvability, and the Response to Selection.

The evolvability of a trait or trait combination quantifies the expected response to selection along a given selection gradient. However, as evolution proceeds, the response to selection may be increasingly constrained by stabilizing selection on correlated traits. Therefore, while evolvabilities may yield good predictions of population divergence over a few generations, evolution over longer timescales may be better predicted by considering the evolvability after accounting for genetic variation bound up in correlations with other traits. The conditional evolvability (*c*) provides a quantitative measure of such constraint and is defined as the expected response along a selection gradient when the non-focal (constraining) traits are held constant (13, 18, 102). Evolvabilities and conditional evolvabilities represent upper and lower limits, respectively, for the response to selection (77).

### Analyses.

#### Univariate analyses.

To identify predictors of population divergence, we defined a divergence study as a set of population means measured as part of the same field survey or common-garden experiment. We paired each divergence estimate with the mean evolvability estimate for each unique trait-species combination. To account for downward bias in the evolvability–divergence relationship due to uncertainty in the predictor variable arising from measurement error and from averaging the evolvability estimates, we corrected the slope by a factor of $1-V_{\mathrm{me}}/V$, where $V_{\mathrm{me}}$ is the measurement (i.e., estimation) error variance in the evolvabilities (variance among repeated evolvability estimates for the same trait) and V is the variance in the predictor variable (i.e., among the evolvabilities) (28).

To estimate the evolvability–divergence relationship, we fit a meta-analytical model including the inverse measurement error variance for each estimate of *d* as weights. We computed the measurement-error variance of *d* as $\sigma_{m}^{2}\left(d\right)={2d}^{2}/(n+2)$ (103). We also included the log-transformed number of populations and the log-transformed maximum geographic distance between populations as fixed covariates, and random intercepts for species and divergence-study identity. To estimate variation in slopes across divergence studies, we included a random-slope term for evolvability per divergence study. We fit the model with the glmmTMB R package (104).

To assess whether the evolvability–divergence relationship varied depending on trait groups (floral vs. vegetative traits), mating systems (selfing vs. mixed-mating vs. outcrossing species), or study environment for the divergence data (greenhouse, outdoor common garden, field), we fit additional models similar to the baseline model, but including the interaction between evolvability and the focal moderator variable. We assessed statistical support for slope heterogeneity by comparing AIC values of the baseline model and the more complex models (with ΔAIC > 2 indicating a detectable difference).

#### Multivariate analyses.

Patterns of evolvability and divergence for multivariate phenotypes can be summarized by variance matrices. We will refer to the mean-scaled within-population genetic variance matrix as **G**, and the proportional (ln-scale) among-population variance matrix as **D**. The benefit of a multivariate analysis is that trait correlations may generate directions (axes of phenotypic variation) of high and low evolvability that are not apparent from univariate analyses. For example, consider two traits that have the same genetic variance, but that are positively correlated genetically. The direction of the highest evolvability will be along the 1:1 line of the two traits and the direction of the lowest evolvability will be perpendicular to this. In this case we would not find any relationship between evolvability and divergence in a univariate analysis (because there would be no variation in evolvability), while a multivariate analysis could reveal one.

We studied multivariate evolvability–divergence relationships for a set of “cases” where paired G- and D-matrices for at least three traits were available. The case studies included both original analyses based on raw data (13 cases, *SI Appendix*, Appendix 1), and analyses based on summary statistics (G-matrices and matrices of population means, 27 cases). Our approach to estimating the matrices is described in *SI Appendix*, Appendix 6, and the R code implementing each analysis is available at https://github.com/oysteiop/EvolDiv. A given G- or D-matrix could be part of several cases, if, e.g., several G-matrices were available for the same study system (*SI Appendix*, Appendix 1). For each case, we defined focal phenotypic directions (trait combinations) using the eigenvectors, **v**, of both **G** and **D** (“G-directions” and “D-directions” hereafter). Eigenvectors represent uncorrelated orthogonal directions, and eigenvectors of the G- and D-matrices maximizes the variation in evolvability and divergence, respectively. The evolvability (predicted response to selection) along a unit-length eigenvector is given by *e*(**v**) = **v**^T^**Gv** (13), where ^T^ denotes transposition. The conditional evolvability along this vector is *c*(**v**) =(v ^T^**G**^−1^**v**)^−1^, where ^−1^ denotes the inverse. For the orthogonal eigenvectors of **G**, *c*(**v**) = *e*(**v**). We computed the population divergence (among-population variance) along the eigenvectors as *d*(**v**) = **v**^T^**Dv**.

We estimated the evolvability–divergence relationships by regressing *d*(**v**) on *e*(**v**), which is directly analogous to the univariate analyses. To quantify uncertainty, we first derived the sampling distributions for **G** and **D**. For the error-corrected D-matrices estimated from summary statistics and the D-matrices estimated from raw data, we used the posterior distributions from the Bayesian mixed models. For the G-matrices, we followed the Monte Carlo simulation approach of Noble et al. (47), in which we resampled the data from the multivariate normal distribution $X_{\mathrm{ij}}∼MVN\left(\bar{x},\;G\right)$, where *i* is the number of drawn samples for each trait *j*, $\bar{x}$ is the vector of trait means, and G is the focal G-matrix. We set the number of samples to the number of families in the original study (i.e., the effective sample size of the breeding designs), and the vector of trait means $\bar{x}$ to 1 (i.e., the mean after mean-scaling). From this, we calculated the simple among-trait variance matrix of the resampled data $X_{\mathrm{ij}}$ for each of 1,000 iterations to derive a sampling distribution for each G-matrix. Finally, we estimated the SE of each evolvability–divergence slope as the SD of the regression slopes based on randomly paired **G**s and **D**s drawn from their respective sampling distributions.

To estimate the mean slope of divergence on evolvability weighed by the uncertainty in the estimates, we fit a meta-analytical model including the inverse sampling variance for each slope estimate as weights. We further included random intercepts for study species and D-matrix identity (nested in species). This approach accounts for some sources of error in the slopes of the evolvability–divergence relationships, though we note that these analyses are further complicated by estimation error in the matrices because this leads to an upward bias in the leading eigenvalue and a downward bias in the trailing eigenvalue (105). Consequently, the regression fitted for the D**-**directions is upwardly biased due to systematic error along the y-axis, while the regression for the G-directions is downwardly biased due to systematic error along the x-axis (106, 107). An alternative approach is to consider the directions represented by the eigenvectors of the phenotypic covariance matrix **P**, which will be unbiased assuming that estimation error in **P** is independent of estimation error in **G** and **D**. We included this approach for those cases where P-matrices were available or could be estimated for one or more populations (when more than one estimate was available, we used the mean).

#### Evolvability along observed divergence vectors.

The above analyses focus on evolvability–divergence relationships across traits within a G-matrix. If genetic constraints are important, we also expect populations to have diverged along phenotypic directions associated with above-average evolvability (21). To assess whether this was the case, we computed divergence vectors from each focal population (in which a G-matrix was estimated) to each non-focal population as $\Delta {\bar{x}}_{\log}=log\left({\bar{x}}_{1}\right)-log\left({\bar{x}}_{0}\right)$. These divergence vectors describe the realized evolution of the multivariate phenotype between the two populations. Following Hansen and Houle (13), we computed the evolvability along $\Delta {\bar{x}}_{\log}$ normalized to unit length as *e*($\Delta {\bar{x}}_{\log}$) = $\Delta {\bar{x}}_{\log}$^T^**G**$\Delta {\bar{x}}_{\log}$, the conditional evolvability as *c*($\Delta {\bar{x}}_{\log}$) = ($\Delta {\bar{x}}_{\log}$^T^**G**^−1^$\Delta {\bar{x}}_{\log}$)^−1^, and proportional evolutionary divergence of each population as $d\left(\;\Delta {\bar{x}}_{log}\;\right)\;=\;E\left[\;\left|\Delta {\bar{x}}_{\log}\right|\;\right]$. We then asked whether *e*($\Delta {\bar{x}}_{\log}$) and *c*($\Delta {\bar{x}}_{\log}$) were greater than the average evolvability and conditional evolvability, respectively, of the focal-population G-matrix. To estimate the mean ratios *e*($\Delta {\bar{x}}_{\log}$)/$\bar{e}$ and *c*($\Delta {\bar{x}}_{\log}$)/$\bar{c}$, we obtained sampling variances through the same resampling method as above and fit a meta-analytical model including inverse sampling variances as weights, and random intercepts for G-matrix and population identity. Finally, we explored graphically the relationship between *d*($\Delta {\bar{x}}_{\log}$), *e*($\Delta {\bar{x}}_{\log}$) and *c*($\Delta {\bar{x}}_{\log}$) for each divergence study (i.e., a set of population means combined with a G-matrix), to ask whether populations diverging in directions of greater evolvability tend to have diverged farther from the focal population.

## Supplementary Material

Appendix 01 (PDF)

## Data Availability

Databases of evolvability and divergence, analysis code data have been deposited in [github.com/oysteiop/EvolDiv] (10.5281/zenodo.7383085) (108).
