# Supplementary material for: Evolvability and trait function predict phenotypic divergence of plant populations
Source: Proc Natl Acad Sci U S A. 2022 Dec 29;120(1):e2203228120. doi: 10.1073/pnas.2203228120 (PMC9910613; doi:10.1073/pnas.2203228120)
Supplement: Supplementary file 1 — Appendix 01 (PDF) [file pnas.2203228120.sapp.pdf]

PNAS

## Supporting Information for

# Evolvability and trait function predict phenotypic divergence of plant populations

Øystein H. Opedal[1,*], W. Scott Armbruster[2,3], Thomas F. Hansen[4], Agnes Holstad[5], Christophe Pélabon[5], Stefan Andersson[1], Diane R. Campbell[6], Christina M. Caruso[7], Lynda F. Delph[8], Christopher G. Eckert[9], Åsa Lankinen[10], Greg M. Walter[11], Jon Ågren[12] & Geir H. Bolstad[13]

[1]Department of Biology, Biodiversity Unit, Lund University, 223 62 Lund, Sweden
[2]School of Biological Sciences, King Henry Building, King Henry I Street, University of Portsmouth, Portsmouth PO1 2DY, UK
[3]Institute of Arctic Biology, University of Alaska, Fairbanks, AK 99775, USA
[4]Centre for Ecological and Evolutionary Synthesis, Department of Biology, University of Oslo, Oslo 0316, Norway
[5]Centre for Biodiversity Dynamics, Department of Biology, Norwegian University of Science and Technology, Trondheim 7491, Norway
[6]Department of Ecology and Evolutionary Biology, University of California, Irvine, CA 92697, USA
[7]Department of Integrative Biology, University of Guelph, Guelph, Ontario N1G 2W1 Canada
[8]Department of Biology, Indiana University, Bloomington, IN 47405, USA
[9]Department of Biology, Queen's University, Kingston, Ontario K7L 3N6 Canada
[10]Department of Plant Protection Biology, Swedish University of Agricultural Sciences, P.O. Box 190, 234 22 Lomma, Sweden
[11]School of Biological Sciences, Monash University, Melbourne 3800, Australia
[12]Department of Ecology and Genetics, Evolutionary Biology Centre, Uppsala University, Norbyvägen 18 D, 753 26 Uppsala, Sweden
[13]Norwegian Institute for Nature Research (NINA), Trondheim 7485, Norway

*Corresponding author e-mail: oystein.opedal@biol.lu.se

## This PDF file includes:

**Appendix 1. Lists of trait categories, divergence studies and G-matrices**
Table S1. List of floral trait categories in the evolvability database, with examples of traits.

Table S2. List of vegetative trait categories in the evolvability database, with examples of traits.

Table S3. List of divergence studies.

Table S4. List of G-matrices included in the multivariate evolvability-divergence analyses.

**Appendix 2. Assessing the dimensionality/trait heterogeneity problem**
Fig. S1. Comparison of evolvabilities and proportional population divergence for traits belonging to different dimension classes.

Fig. S2. Univariate relationship between evolvability and population divergence (ln-scaled among-population variances) for traits belonging to different dimension classes.

Fig. S3. Univariate relationship between evolvability and population divergence (ln-scaled among-population variances) after removing the effect of dimensionality statistically by taking residuals of models on the form divergence ~ dimension and evolvability ~ dimension.

Fig. S4. Evolvability-divergence patterns for studies containing different numbers and combinations of trait dimensions.

Fig. S5. Reduced version of Fig. 4 of the main text, including only cases where all traits in the G- and D-matrices are linear size measures.

Fig. S6. Reduced version of Fig. 5 in the main text, including only cases where all traits in the G- and D-matrices are linear size measures.

**Appendix 3. Comparison of evolvability-divergence relationships for unconditional and conditional evolvabilities**

Fig. S7. Comparison of evolvability-divergence relationships estimated using unconditional and conditional evolvabilities.

**Appendix 4. Supplementary figures and extended comparison of trait groups, mating systems, and study environments**

Fig. S8. Proportional population divergence of vegetative (green boxes) life-history (brown boxes) and floral (blue boxes) traits.

Fig. S9. Ratios of proportional population divergence to evolvability for vegetative (green boxes) and floral (blue boxes) traits.

Fig. S10. Proportional population divergence of vegetative and floral traits in selfing, mixed-mating, and outcrossing species.

Fig. S11. Evolvability-divergence patterns for studies including different combinations of trait types.

Fig. S12. Evolvability-divergence patterns for selfing (S), mixed-mating (M) and outcrossing (O) species.

Fig. S13. Evolvability-divergence patterns when the populations scored for divergence were studied in the field, in outside common gardens, and in the greenhouse.

**Appendix 5. Comparison of approaches and comparison to angle approaches**

Fig. S14. Relationship, across divergence studies, between mean proportional evolvabilities along divergence vectors and the slope of the evolvability-divergence relationship for the G-directions.

Fig. S15. Comparison of the evolvability along a divergence vector and the angle between the divergence vector and the leading eigenvector of the G-matrix ($\mathbf{g}_{max}$).

Fig. S16 Comparison of the angle between the leading eigenvectors of the G- and D-matrices, the evolvability-divergence relationship estimated for the G-directions, and the proportional evolvability along a divergence vector.

**Appendix 6. Extended analytical methods**

**SI References**

## Appendix 1. Lists of trait categories, divergence studies and G-matrices

Table S1. List of floral trait categories in the evolvability database, with examples of traits. From Opedal (2019).

| Trait category | Definition | Sub-category examples | Trait examples |
|---|---|---|---|
| Advertisement | Other pollinator-advertisement traits | Pattern | Number of floral spots |
| Allocation | Proportional allocation | Male-female, reproduction | Anther-ovule ratio, capsule-to-plant weight |
| Display | Floral display size | Flower number, inflorescence size | Flower number, inflorescence length |
| Fecundity | Measures of male or female fecundity | Male fecundity, female fecundity | Pollen number, ovule number |
| Female | Female structures not directly influencing pollinator fit | Ovary, stigma | Ovary length, stigma width |
| Fit | Traits affecting accuracy of pollen transfer | Tube length, male | Corolla tube length, stamen length |
| Fitness | Fitness components | Seed size, fruit set | Seed diameter, capsule number |
| Flower size | Size of individual flowers | Corolla, petal | Corolla diameter, petal area |
| Herkogamy | Spatial separation of male and female sexual structures | Herkogamy | Anther-stigma distance |
| Inflorescence | Inflorescence traits not directly measuring floral display size | Inflorescence | Peripheral achenes per head |
| Male | Male structures not directly influencing pollinator fit | Anther, pollen | Anther size, pollen size |
| Ontogeny | Flower or blossom development | Female phase | Duration of female phase |
| Pedicel | Length of floral pedicel | Pedicel length | Pedicel length |
| Peduncle | Length of floral peduncle | Peduncle length | Peduncle length |
| Reward | Amount or rate of reward secretion | Nectar, resin | Nectar sugar concentration, nectar secretion rate |
| Shape | Floral trait shapes and ratios | Flower shape | Corolla length by width |

Table S2. List of vegetative trait categories in the evolvability database, with examples of traits. From Opedal (2019).

| Trait category | Definition | Sub-category examples | Trait examples |
|---|---|---|---|
| Cotyledon size | Size of cotyledons | Cotyledon diameter | Cotyledon diameter |
| Defence | Vegetative defence traits | Trichome | Trichome density |
| Functional | Trait related to physiological function | Specific leaf area | Specific leaf area |
| Growth | Measures of increase in biomass or size | Height | Height at 10 weeks |
| Leaf number | Number of leaves | Leaf number | Leaf number |
| Leaf size | Size of individual leaves | Leaf size | Leaf length, leaf area |
| Leaf thickness | Thickness of leaves | Leaf thickness | Leaf thickness |
| Plant size | Measures of overall vegetative size | Height, mass | Plant height, biomass |
| Pubescence | Traits related to leaf pubescence | Pubescence | Hair density |
| Rosette size | Size of basal leaf rosette | Rosette size | Rosette diameter |
| Shape | Vegetative trait shapes and ratios | Plant shape | Branch length by height |

Table S3. List of divergence studies (sets of population means measured during the same field survey or common-garden study). The given references describe the study systems, traits, and the data collection methods.

| Species | Pops. | Traits | Environment | Reference |
|---|---|---|---|---|
| *Aquilegia pyrenaica* | 7 | 8 | field | Alcántara et al. 2010 |
| *Aquilegia pyrenaica* | 7 | 6 | field | Castellanos et al. 2011 |
| *Aquilegia vulgaris* | 8 | 8 | field | Alcántara et al. 2010 |
| *Aquilegia vulgaris* | 8 | 6 | field | Castellanos et al. 2011 |
| *Nigella degenii* | 2 | 6 | greenhouse | Andersson 1997 |
| *Crepis tectorum* | 54 | 5 | greenhouse | Andersson 1991 |
| *Fragaria virginiana* | 3 | 4 | field | Ashman 2003 |
| *Turnera ulmifolia* | 10 | 3 | greenhouse | Barrett and Shore 1987 |
| *Aquilegia canadensis* | 7 | 4 | field | Bartkowska et al. 2018 |
| *Aquilegia canadensis* | 5 | 3 | field | Herlihy and Eckert 2007 |
| *Holcus lanatus* | 2 | 6 | greenhouse | Billington et al. 1988 |
| *Dalechampia scandens A* | 9 | 22 | greenhouse | Bolstad et al. 2014 |
| *Dalechampia scandens A* | 3 | 24 | greenhouse | Hansen et al. 2003 |
| *Dalechampia scandens A* | 15 | 14 | field | Opedal et al. 2016 |
| *Dalechampia scandens A* | 12 | 18 | greenhouse | Opedal et al. 2016 |
| *Dalechampia scandens B* | 12 | 5 | greenhouse | Bolstad et al. 2014 |
| *Medicago truncatula* | 2 | 24 | greenhouse | Bonnin et al. 1997 |
| *Ipomopsis aggregata* | 2 | 3 | field | Campbell et al. 2018 |
| *Ipomopsis aggregata* | 3 | 4 | field | Campbell et al. 2018 |
| *Ipomopsis aggregata* | 7 | 5 | field | Caruso 2000 |
| *Ipomopsis aggregata* | 5 | 4 | field | Caruso 2001 |
| *Ipomopsis aggregata x tenuituba* | 7 | 3 | field | Campbell et al. 2018 |
| *Ipomopsis tenuituba* | 3 | 3 | field | Campbell et al. 2018 |
| *Mimulus guttatus* | 2 | 9 | greenhouse | Carr and Fenster 1994 |
| *Mimulus guttatus* | 2 | 4 | greenhouse | Fenster and Carr 1997 |
| *Mimulus micranthus* | 2 | 9 | greenhouse | Carr and Fenster 1994 |
| *Mimulus micranthus* | 2 | 4 | greenhouse | Fenster and Carr 1997 |
| *Talinum mengesii* | 10 | 5 | greenhouse | Carter and Murdy 1986 |
| *Talinum teretifolium* | 10 | 6 | greenhouse | Carter and Murdy 1986 |
| *Lobelia siphilitica* | 2 | 6 | greenhouse | Caruso 2004 |
| *Lobelia siphilitica* | 10 | 9 | greenhouse | Caruso 2012 |
| *Lobelia siphilitica* | 3 | 7 | field | Caruso et al. 2003 |
| *Lobelia cardinalis* | 3 | 7 | field | Caruso et al. 2003 |
| *Collinsia heterophylla* | 4 | 4 | field | Charlesworth and Mayer 1995 |
| *Collinsia heterophylla* | 4 | 5 | greenhouse | Charlesworth and Mayer 1995 |
| *Collinsia heterophylla* | 13 | 4 | greenhouse | Lankinen et al. 2017 |
| *Lythrum salicaria* | 20 | 12 | greenhouse | Colautti and Barrett 2011 |
| *Spergularia marina* | 4 | 9 | greenhouse | Delesalle and Mazer 1995 |
| *Solanum carolinense* | 3 | 5 | greenhouse | Elle 1998 |
| *Ambrosia artemisiifolia* | 6 | 4 | common garden | McGoey and Stinchcombe 2021 |
| *Clarkia dudleyana* | 11 | 6 | greenhouse | Podolsky et al. 1997 |
| *Arabidopsis lyrata* | 4 | 4 | common garden | Puentes et al. 2016 |
| *Heterosperma pinnatum* | 6 | 17 | greenhouse | Venable and Burquez 1989 |

| | | | | |
|---|---|---|---|---|
| *Senecio pinnatifolius* | 16 | 10 | greenhouse | Walter et al. 2018 |
| *Senecio integrifolius* | 2 | 25 | common garden | Widen and Andersson 1993 |
| *Brassica cretica* | 5 | 7 | greenhouse | Widen et al. 2002 |
| *Eichhornia paniculata* | 2 | 3 | greenhouse | Worley and Barrett 2001 |
| *Silene latifolia* | 3 | 2 | greenhouse | Yu et al. 2011 |

Table S4. List of G-matrices included in the multivariate evolvability-divergence analyses. The column 'Type' indicate whether we used a published G-matrix or worked with raw data (Original). G-matrices marked with an asterisk (*) were included only in the divergence vector analyses.

| Species | Population | Traits | Type | Reference |
|---|---|---|---|---|
| *Lobelia siphilitica* | CERA | 5 | Published | Caruso 2004 |
| *Lobelia siphilitica* | Krumm | 5 | Published | Caruso 2004 |
| *Aquilegia canadensis* | QFP1 | 3 | Published | Herlihy and Eckert 2007 |
| *Aquilegia canadensis* | QLL3 | 3 | Published | Herlihy and Eckert 2007 |
| *Brassica cretica* | Go | 7 | Published | Widen et al. 2002 |
| *Brassica cretica* | Ma | 7 | Published | Widen et al. 2002 |
| *Brassica cretica* | Mk | 7 | Published | Widen et al. 2002 |
| *Brassica cretica* | Ro | 7 | Published | Widen et al. 2002 |
| *Brassica cretica* | To | 7 | Published | Widen et al. 2002 |
| *Spergularia marina* | ASC | 6 | Published | Delesalle and Mazer 1995 |
| *Spergularia marina* | COP | 6 | Published | Delesalle and Mazer 1995 |
| *Spergularia marina* | MSH | 6 | Published | Delesalle and Mazer 1995 |
| *Spergularia marina* | SMB | 6 | Published | Delesalle and Mazer 1995 |
| *Solanum carolinense* | Landfill | 5 | Published | Elle 1998 |
| *Solanum carolinense* | Old Field | 5 | Published | Elle 1998 |
| *Solanum carolinense* | Sheep Pasture | 5 | Published | Elle 1998 |
| *Clarkia dudleyana* | Tanbark Flats (124) | 4 | Published | Podolsky et al. 1997 |
| *Ipomopsis aggregata* | Vera Falls | 4 | Published | Campbell 1996 |
| *Turnera ulmifolia* | A20 | 3 | Published | Shore and Barrett 1990 |
| *Fragaria virginiana* | Crawford County | 4 | Published | Ashman 2003 |
| *Mimulus guttatus** | S | 7 | Published | Carr and Fenster 1994 |
| *Mimulus guttatus** | T | 7 | Published | Carr and Fenster 1994 |
| *Mimulus micranthus** | 301 | 7 | Published | Carr and Fenster 1994 |
| *Mimulus micranthus** | 305 | 7 | Published | Carr and Fenster 1994 |
| *Nigella degenii** | Mikonos | 4 | Published | Andersson 1997 |
| *Nigella degenii** | Siros | 5 | Published | Andersson 1997 |
| *Dalechampia scandens A* | Tulum | 6 | Original | Hansen et al. 2003 |
| *Dalechampia scandens B* | Tovar | 5 | Original | Bolstad et al. 2014 |
| *Crepis tectorum* | Vickleby | 5 | Original | Andersson and Ofori 2013 |
| *Senecio pinnatifolius* | Dune | 10 | Original | Walter et al. 2018 |
| *Senecio pinnatifolius* | Head | 10 | Original | Walter et al. 2018 |
| *Senecio pinnatifolius* | Table | 10 | Original | Walter et al. 2018 |
| *Senecio pinnatifolius* | Wood | 10 | Original | Walter et al. 2018 |
| *Arabidopsis lyrata* | SPIT | 4 | Original | Puentes et al. 2016 |
| *Arabidopsis lyrata* | STUC | 4 | Original | Puentes et al. 2016 |
| *Arabidopsis lyrata* | STUS | 4 | Original | Puentes et al. 2016 |
| *Arabidopsis lyrata* | VIS | 4 | Original | Puentes et al. 2016 |
| *Silene latifolia** | 3 pops | 2 | Original | Yu et al. 2011 |
| *Lythrum salicaria* | 20 pops | 7 | Original | Colautti and Barrett 2011 |

**References (Appendix 1)**

Alcántara JM, Bastida JM, Rey PJ. 2010. Linking divergent selection on vegetative traits to environmental variation and phenotypic diversification in the Iberian columbines (*Aquilegia*). Journal of Evolutionary Biology 23: 1218-1233.

Andersson S. 1991. Geographical variation and genetic analysis of leaf shape in *Crepis tectorum* (Asteraceae). Plant Systematics and Evolution 178: 247-258.

Andersson S. 1997. Genetic constraints on phenotypic evolution in *Nigella* (Ranunculaceae). Biological Journal of the Linnean Society 62: 519-532.

Andersson, S., and J. K. Ofori. 2013. Effects of mating system on adaptive potential for leaf morphology in *Crepis tectorum* (Asteraceae). Annals of Botany 112:947-955.

Ashman TL. 2003. Constraints on the evolution of males and sexual dimorphism: field estimates of genetic architecture of reproductive traits in three populations of gynodioecious *Fragaria virginiana*. Evolution 57: 2012-2025.

Barrett SCH, Shore JS. 1987. Variation and evolution of breeding systems in the *Turnera ulmifolia* L. complex (Turneraceae). Evolution 41: 340-354.

Bartkowska MP, Wong AC, Sagar SP, Zeng L, Eckert CG. 2018. Lack of spatial structure for phenotypic and genetic variation despite high self-fertilization in *Aquilegia canadensis* (Ranunculaceae). Heredity (Edinb) 121: 605-615.

Billington HL, Mortimer AM, McNeilly T. 1988. Divergence and genetic structure in adjacent grass populations. I. Quantitative genetics. Evolution 42: 1267-1277.

Bolstad GH, Hansen TF, Pélabon C, Falahati-Anbaran M, Pérez-Barrales R, Armbruster WS. 2014. Genetic constraints predict evolutionary divergence in *Dalechampia* blossoms. Philos Trans R Soc Lond B Biol Sci 369: 20130255.

Bonnin I, Prosperi JM, Olivieri I. 1997. Comparison of quantitative genetic parameters between two natural populations of a selfing plant species, *Medicago truncatula* Gaertn. Theoretical and Applied Genetics 94: 641-651.

Campbell, D. R. 1996. Evolution of floral traits in a hermaphroditic plant: field measurements of heritabilities and genetic correlations. Evolution 50:1442-1453.

Campbell DR, Faidiga A, Trujillo G. 2018. Clines in traits compared over two decades in a plant hybrid zone. Annals of Botany DOI: 10.1093/aob/mcy072.

Carr DE, Fenster CB. 1994. Levels of genetic variation and covariation for *Mimulus* (Scrophulariaceae) floral traits. Heredity 72: 606-618.

Carter MEB, Murdy WH. 1986. Divergence for sexual and asexual reproductive characters in *Talinum mengesii* (Portulacaceae). Bulletin of the Torrey Botanical Club 113: 259-267.

Caruso CM. 2000. Competition for pollination influences selection on floral traits of *Ipomopsis aggregata*. Evolution 54: 1546-1557.

Caruso CM. 2001. Differential selection on floral traits of *Ipomopsis aggregata* growing in contrasting environments. Oikos 94: 295-302.

Caruso, C. M. 2004. The quantitative genetics of floral trait variation in *Lobelia*: Potential constraints on adaptive evolution. Evolution 58:732-740.

Caruso CM. 2012. Sexual dimorphism in floral traits of gynodioecious *Lobelia siphilitica* (Lobeliaceae) is consistent across populations. Botany 90: 1245-1251.

Caruso CM, Maherali H, Mikulyuk A, Carlson K, Jackson RB. 2005. Genetic variance and covariance for physiological traits in *Lobelia*: are there constraints on adaptive evolution? Evolution 59: 826-837.

Caruso CM, Peterson SB, Ridley CE. 2003. Natural selection on floral traits of *Lobelia* (Lobeliaceae): Spatial and temporal variation. American Journal of Botany 90: 1333-1340.

Castellanos MC, Alcántara JM, Rey PJ, Bastida JM. 2011. Intra-population comparison of vegetative and floral trait heritabilities estimated from molecular markers in wild *Aquilegia* populations. Molecular Ecology 20: 3513-3524.

Charlesworth D, Mayer S. 1995. Genetic variability of plant characters in the partial inbreeder *Collinsia heterophylla* (Scrophulariaceae). American Journal of Botany 82: 112-120.

Colautti RI, Barrett SC. 2011. Population divergence along lines of genetic variance and covariance in the invasive plant *Lythrum salicaria* in eastern North America. Evolution 65: 2514-2529.

Delesalle VA, Mazer SJ. 1995. The structure of phenotypic variation in gender and floral traits within and among populations of *Spergularia marina* (Caryophyllaceae). American Journal of Botany 82: 798-810.

Elle E. 1998. The quantitative genetics of sex allocation in the andromonoecious perennial, *Solanum carolinense* (L.). Heredity 80: 481-488.

Fenster CB, Carr DE. 1997. Genetics of sex allocation in *Mimulus* (Scrophulariaceae). Journal of Evolutionary Biology 10: 641-661.

Hansen TF, Pélabon C, Armbruster WS, Carlson ML. 2003. Evolvability and genetic constraint in *Dalechampia* blossoms: components of variance and measures of evolvability. Journal of Evolutionary Biology 16: 754-766.

Herlihy CR, Eckert CG. 2007. Evolutionary analysis of a key floral trait in *Aquilegia canadensis* (Ranunculaceae): genetic variation in herkogamy and its effect on the mating system. Evolution 61: 1661-1674.

Lankinen Å, Madjidian JA, Andersson S. 2017. Geographic variation in floral traits is associated with environmental and genetic differences among populations of the mixed mating species *Collinsia heterophylla* (Plantaginaceae). Botany 95: 121-138.

McGoey BV, Stinchcombe JR. 2021. Introduced populations of ragweed show as much evolutionary potential as native populations. Evol Appl 14: 1436-1449.

Opedal ØH, Albertsen E, Armbruster WS, Pérez-Barrales R, Falahati-Anbaran M, Pélabon C. 2016. Evolutionary consequences of ecological factors: pollinator reliability predicts mating-system traits of a perennial plant. Ecology Letters 19: 1486-1495.

Podolsky RH, Shaw RG, Shaw FH. 1997. Population structure of morphological traits in *Clarkia dudleyana*. II. Constancy of within-population genetic variance. Evolution 51: 1785-1796.

Puentes A, Granath G, Ågren J. 2016. Similarity in G matrix structure among natural populations of *Arabidopsis lyrata*. Evolution 70: 2370-2386.

Shore, J. S., and S. C. H. Barrett. 1990. Quantitative genetics of floral characters in homostylous *Turnera ulmifolia* var. *angustifolia* Willd. (Turneraceae). Heredity 64:105-112.

Venable DL, Burquez A. 1989. Quantitative genetics of size, shape, life-history, and fruit characteristics of the seed-heteromorphic composite *Heterosperma pinnatum*. 1. Variation within and among populations. Evolution 43: 113-124.

Walter GM, Aguirre JD, Blows MW, Ortiz-Barrientos D. 2018. Evolution of genetic variance during adaptive radiation. The American Naturalist: E000-E000.

Widén B, Andersson S. 1993. Quantitative genetics of life-history and morphology in a rare plant, *Senecio integrifolius*. Heredity 70: 503-514.

Widén B, Andersson S, Rao G-Y, Widén M. 2002. Population divergence of genetic (co)variance matrices in a subdivided plant species, *Brassica cretica*. Journal of Evolutionary Biology 15: 961-970.

Worley AC, Barrett SCH. 2001. Evolution of floral display in *Eichhornia paniculata* (Pontederiaceae): genetic correlations between flower size and number. Journal of Evolutionary Biology 14: 469-481.

Yu Q, Ellen ED, Wade MJ, Delph LF. 2011. Genetic differences among populations in sexual dimorphism: evidence for selection on males in a dioecious plant. Journal of Evolutionary Biology 24: 1120-1127.

## Appendix 2. Assessing the dimensionality/trait heterogeneity problem

Proportional variances depend on trait measurement dimensions, so that e.g. traits measured as areas will be proportionally more variable than are traits represented by linear measurements. In evolvability-divergence analyses, this means that those traits measured as areas will tend to have both greater evolvabilities and greater among-population variances.

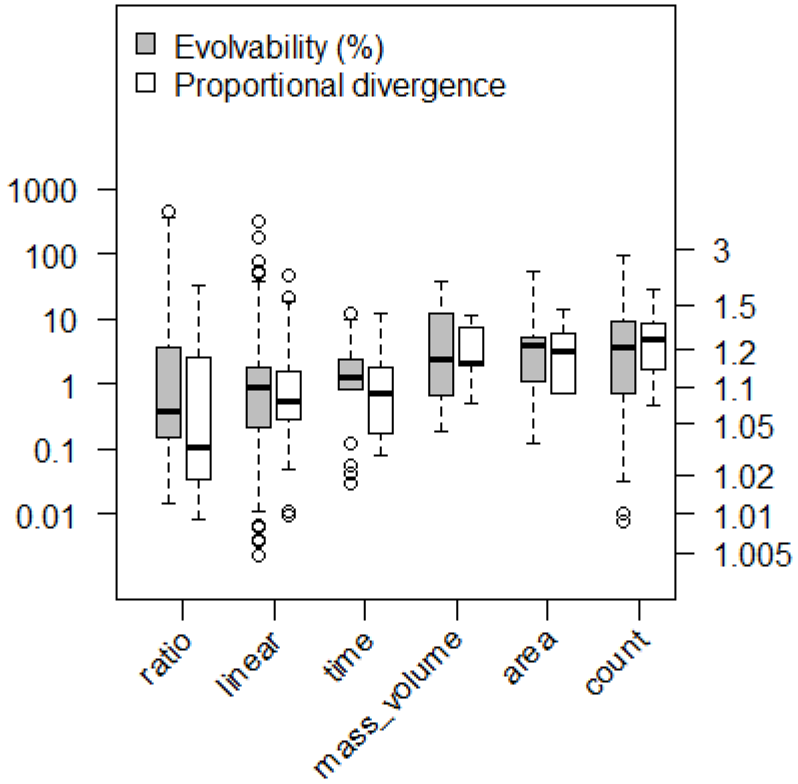


**Figure S1.** Comparison of evolvabilities (grey boxes, left axis) and proportional population divergence (white boxes, right axis) for traits belonging to different dimension classes.

Consequently, if we combine traits of different dimensions in the analysis, a positive relationship can emerge simply as an effect of trait dimensionality. Here, we explore the sensitivity of our analyses to the inclusion of heterogeneous traits (of different dimensions).

As expected, median evolvabilities and median divergences were positively correlated across measurement dimensions (Fig. S2, black dots). The slopes of the evolvability-divergence relationships were similar for those dimension classes with large samples ($n$>38, i.e. linear traits, counts, and ratios), but more variable for the smaller samples ($n$<20) of area traits, mass-volume traits, and time traits. Thus, a model allowing dimension-specific slopes and intercepts was supported statistically over a model with a single slope and intercept ($\Delta$AIC = 126.3).

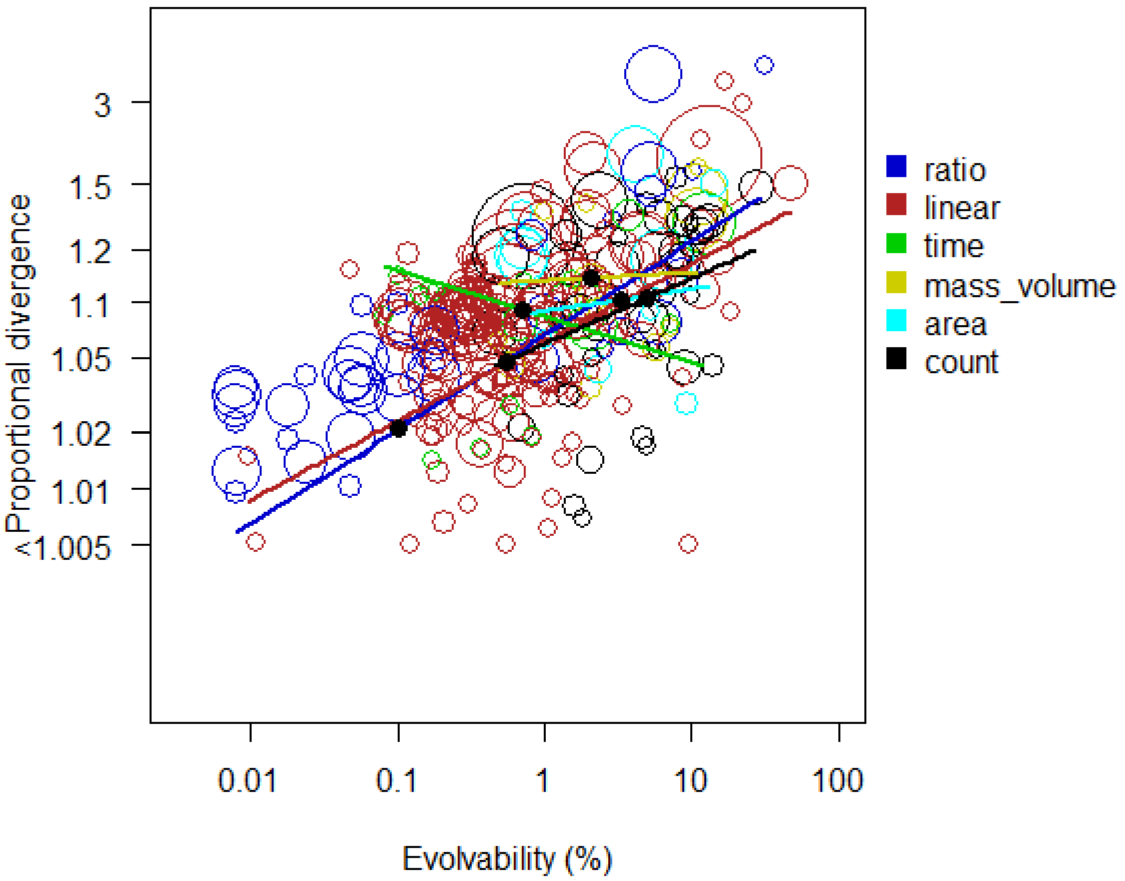


**Figure S2.** Univariate relationship between evolvability and population divergence (ln-scaled among-population variances) for traits belonging to different dimension classes. Circle sizes are proportional to the square root of the number of populations studied. Regression lines show the relationships, and solid dots indicate the expected divergence at the median evolvability in each group. The y-axis gives the proportional divergence of the average population ($d_P$), where a value of 1.1 indicates that the trait mean of an average population has evolved to be c. 10% larger or smaller than the grand mean.

The robustness of the overall evolvability-divergence pattern can also be seen by plotting the relationship between divergence and evolvability after centering both evolvabilities and divergences on the category mean, i.e after 'removing' the dimension effect by taking residuals of models including dimension as a single fixed effect.

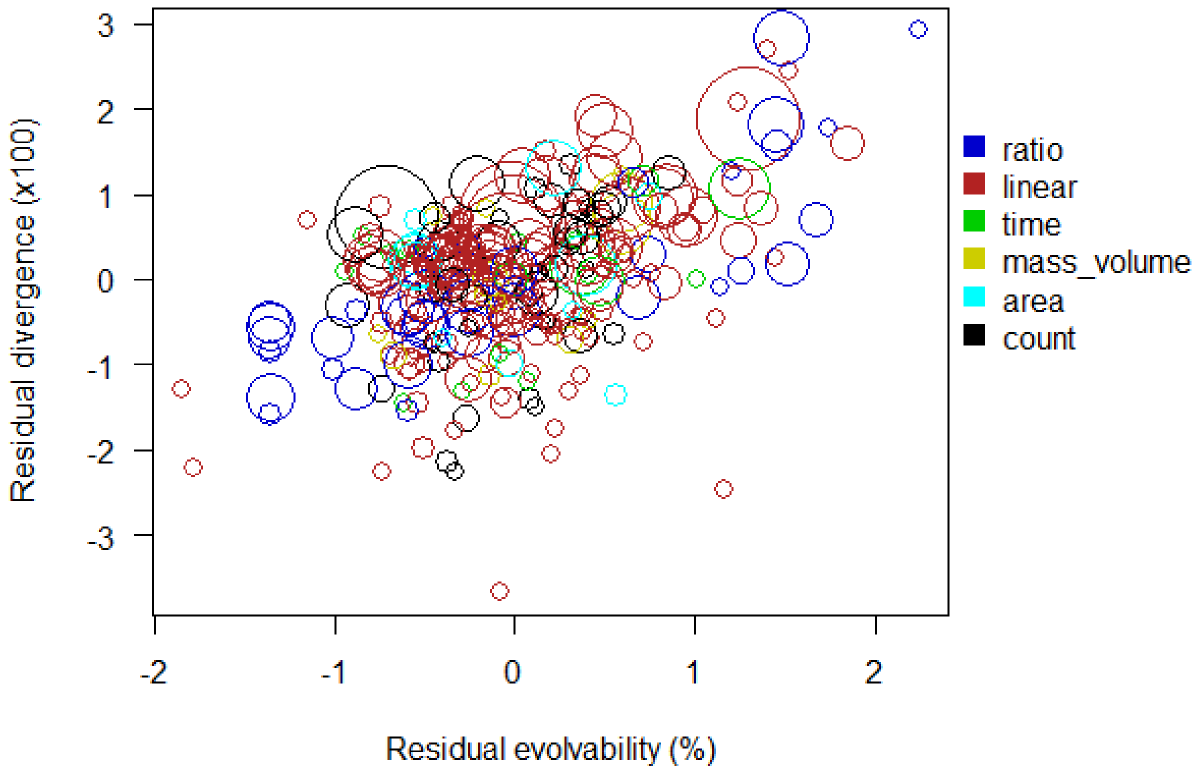


**Figure S3.** Univariate relationship between evolvability and population divergence (ln-scaled among-population variances) after removing the effect of dimensionality statistically by taking residuals of a model on the form divergence ~ dimension. Circle sizes are proportional to the square root of the number of populations studied.

In a model similar to our baseline meta-analytical model (Main text, Table 1), but with residual evolvability as a predictor of residual divergence, the slope was slightly shallower (0.68 ± 0.12), and the variance explained was slightly lower ($r^2_M$ = 29.4%, $r^2_C$ = 83.0).

These analyses suggest that, although dimensionality clearly affect trait variances at all levels, the general positive relationship between evolvability and divergence is not a simple artefact of dimensionality. Furthermore, positive evolvability-divergence relationship have been detected in analyses of highly homogeneous traits such as landmarks on *Drosophila* wings (3), and floral-bract dimensions in *Dalechampia* (2).

In the multivariate analyses, the slopes of the evolvability-divergence relationships for the G-directions were slightly steeper when the traits comprised two dimension-classes, but there was no overall relationship between the slopes and the number of dimension classes. Note also that the linear-only cases fell close to the overall median in all comparisons.

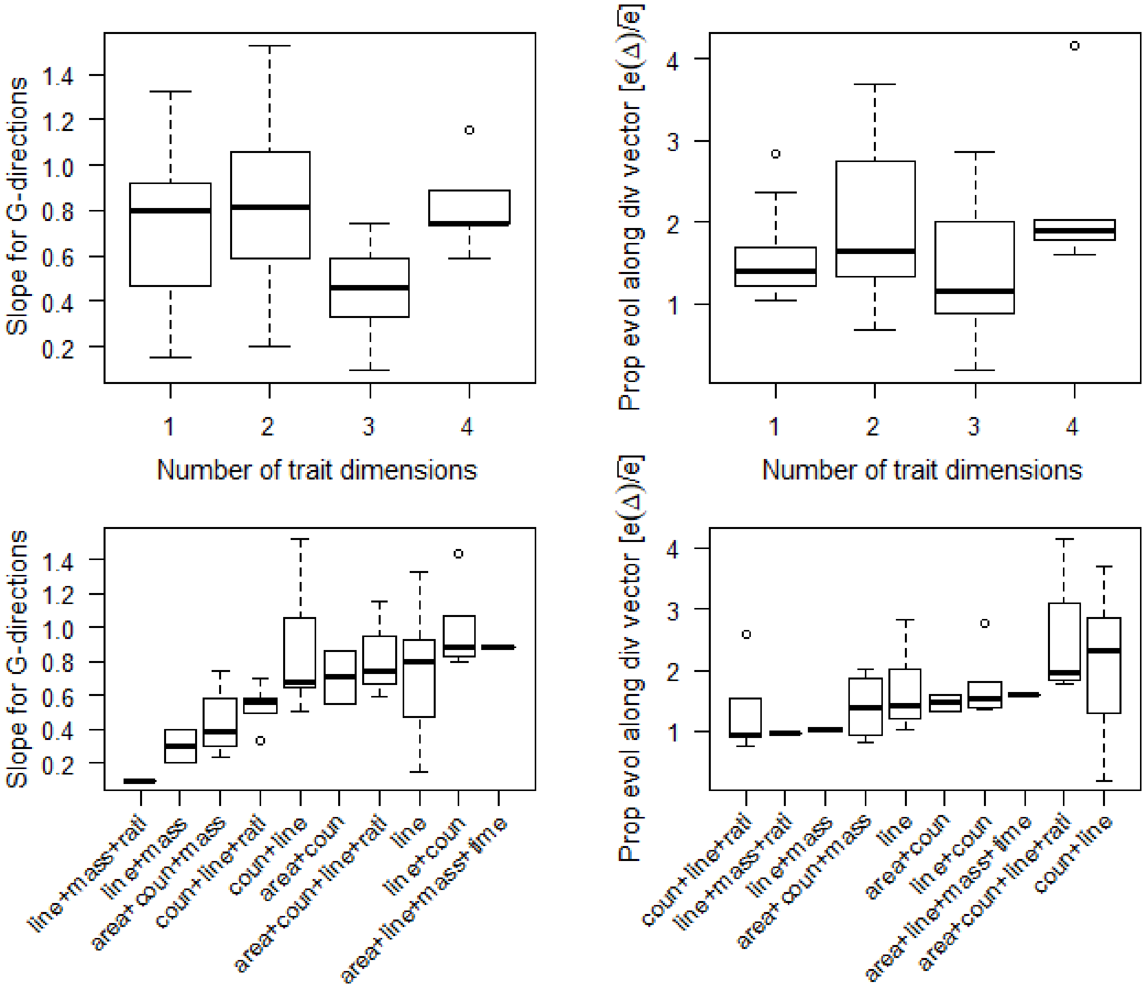


**Figure S4.** Evolvability-divergence patterns for studies containing different numbers and combinations of trait dimensions. Abbreviations: line = linear size measures, mass = mass/volume, rati = ratios, area = area, coun = counts, time = time.

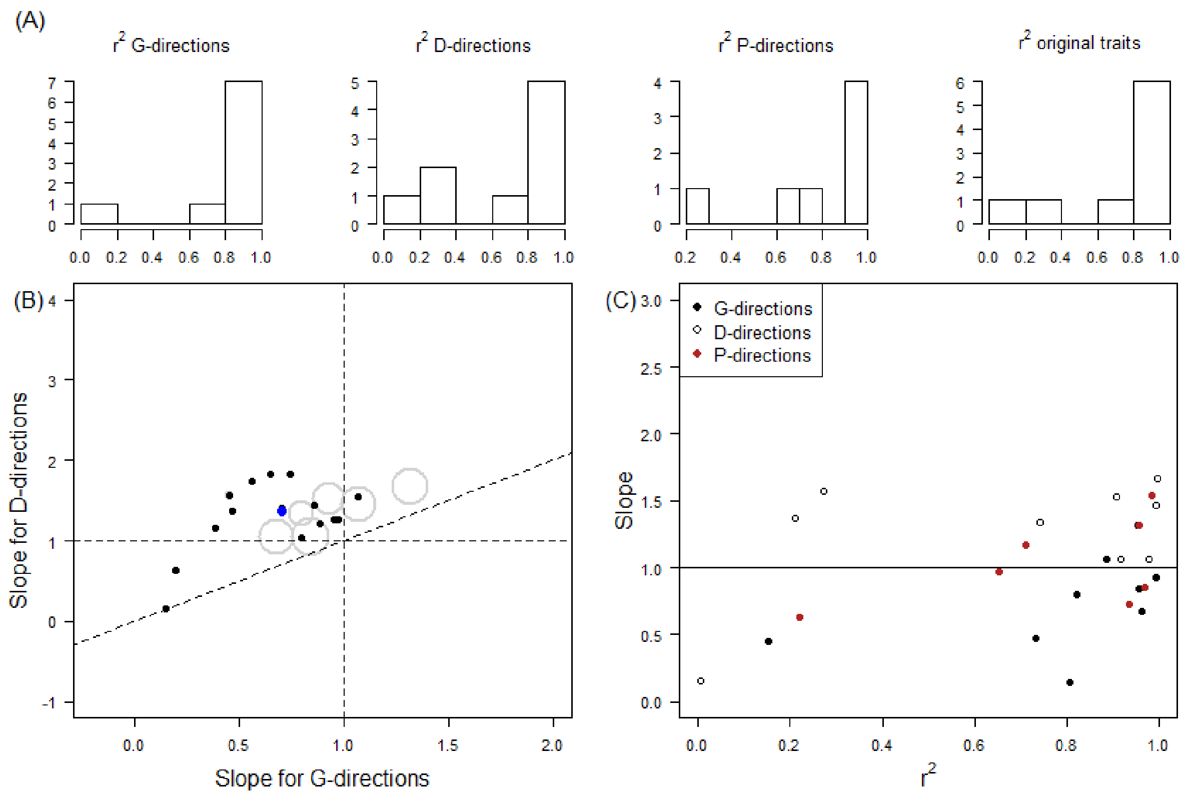


**Figure S5**. Reduced version of Fig. 4 of the main text, including only cases where all traits in the G- and D-matrices are linear size measures.

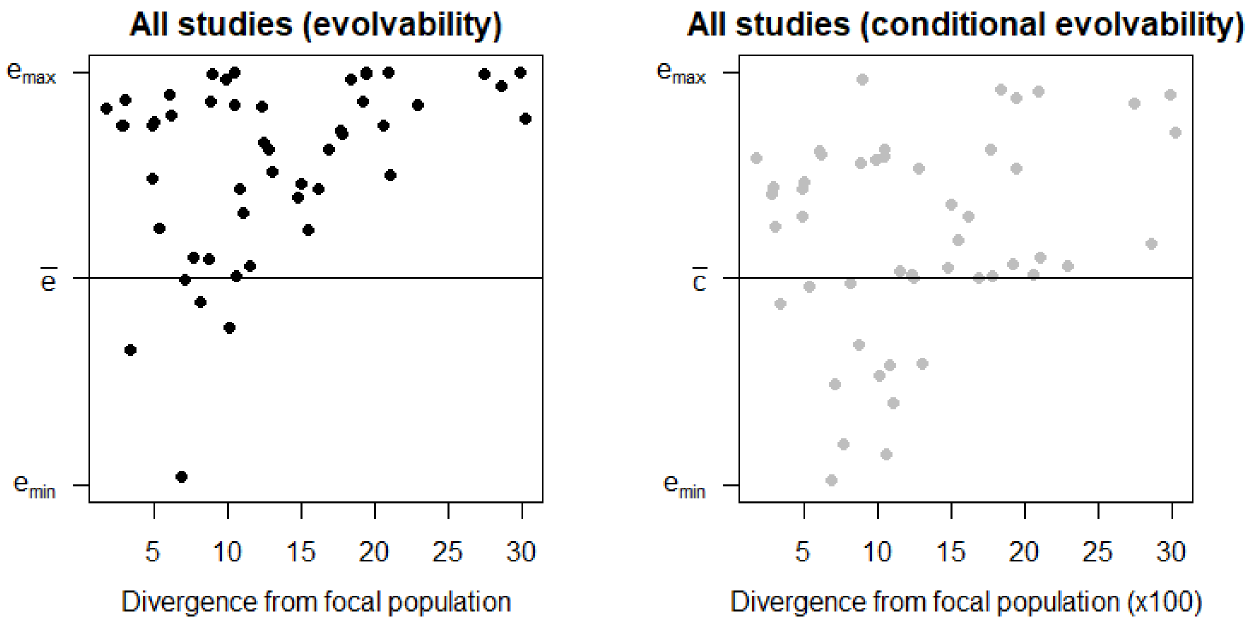


**Figure S6**. Reduced version of summary panels in Fig. 5 in the main text, including only cases where all traits in the G- and D-matrices are linear size measures.

## Appendix 3. Comparison of evolvability-divergence relationships for unconditional and conditional evolvabilities

Here, we explore how the evolvability-divergence relationships change when substituting evolvabilities with conditional evolvabilities. There were no systematic differences in slopes or coefficients of determination ($r^2$) between the evolvability measures. Both slopes and $r^2$ values were about equally likely to increase and decrease when substituting conditional evolvabilities with evolvabilities.

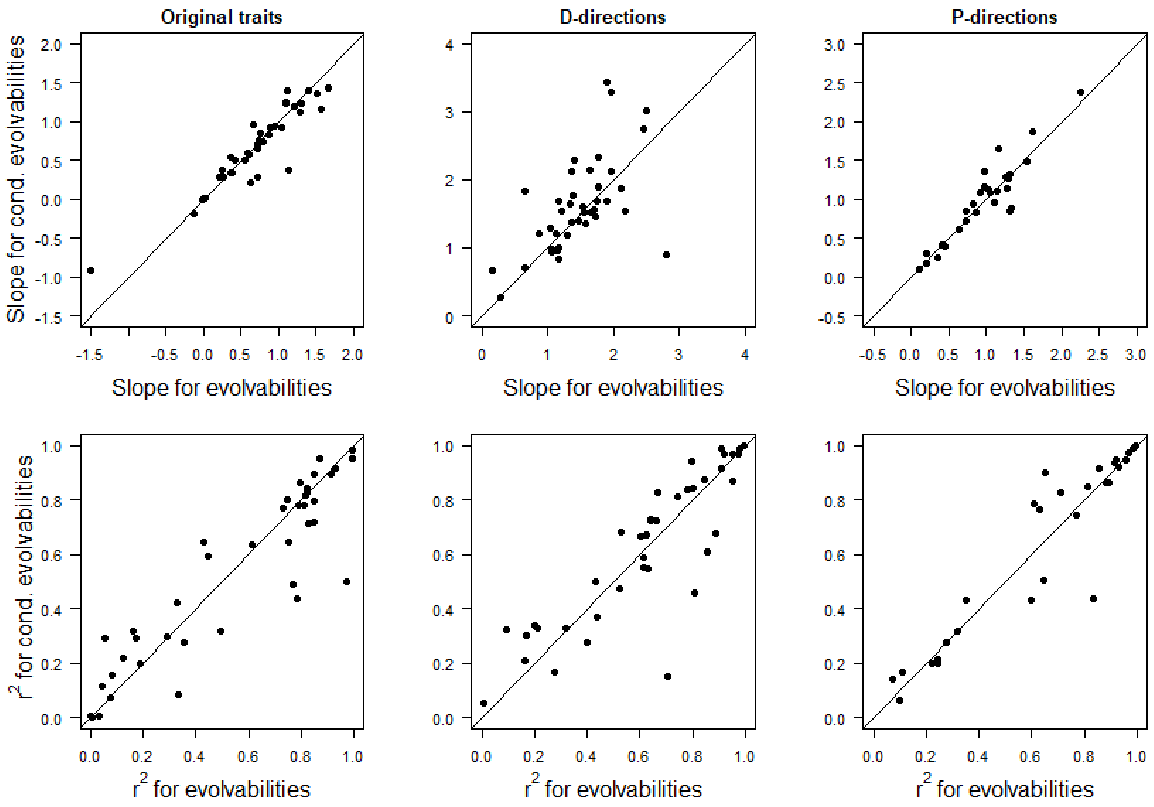


**Figure S7.** Comparison of evolvability-divergence relationships estimated using unconditional and conditional evolvabilities. The conditional evolvabilities for the original traits, the D-directions, and the P-directions are conditional on the entire G-matrix.

## Appendix 4. Supplementary figures and extended comparison of trait groups, mating systems, and study environments

*Patterns of population divergence across trait groups*

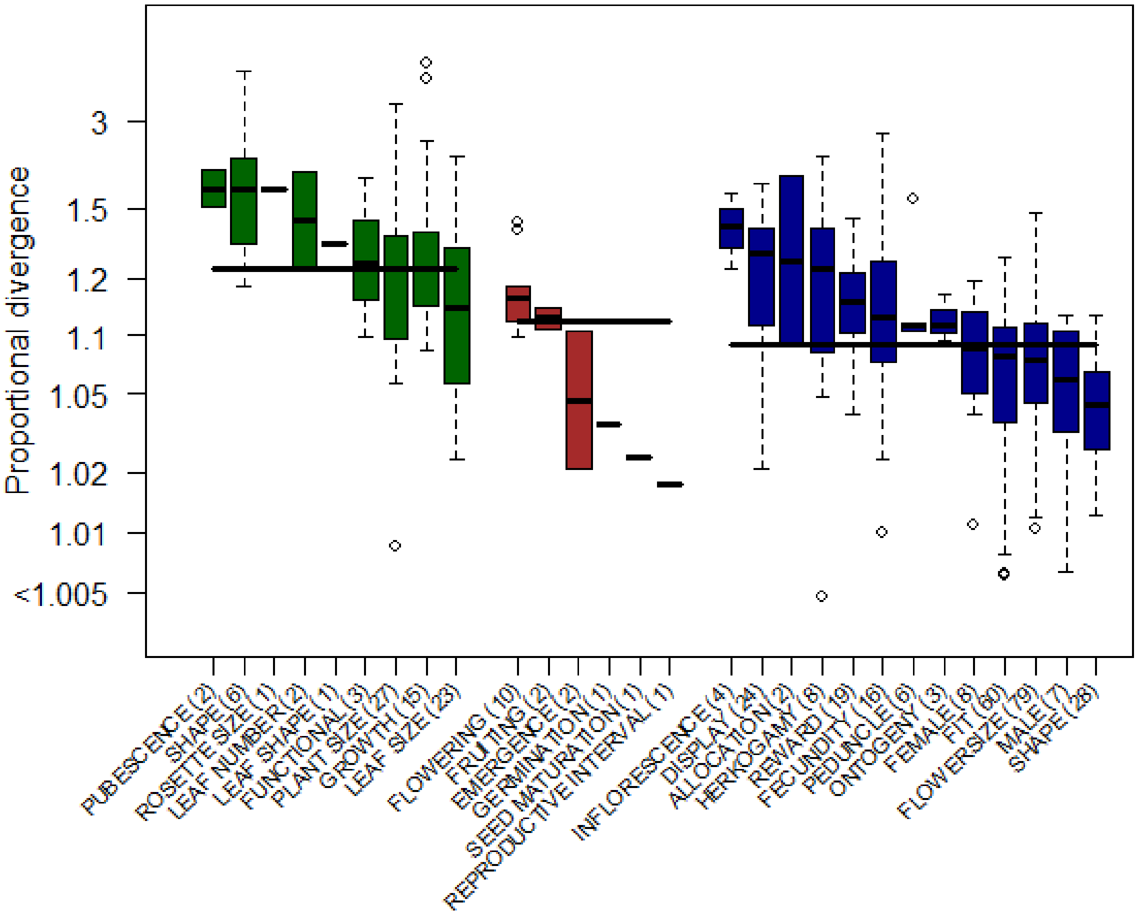


**Figure S8.** Proportional population divergence of vegetative (green boxes) life-history (brown boxes) and floral (blue boxes) traits. The *y*-axis gives the proportional divergence of the average population ($d_P$), where a value of 1.1 indicates that the trait mean of an average population has evolved to be c. 10% larger or smaller than the grand mean. Thick lines across boxes show the median of each trait category, and thick lines within boxes show median values for each trait subcategory. Boxes extend from the first to third quartile, range bars extend to 1.5 times the inter-quartile range, and data points outside this range are shown as open circles. Sample sizes are given in parentheses for each trait subcategory.

*Comparing the ratio of divergence to evolvability across trait groups*

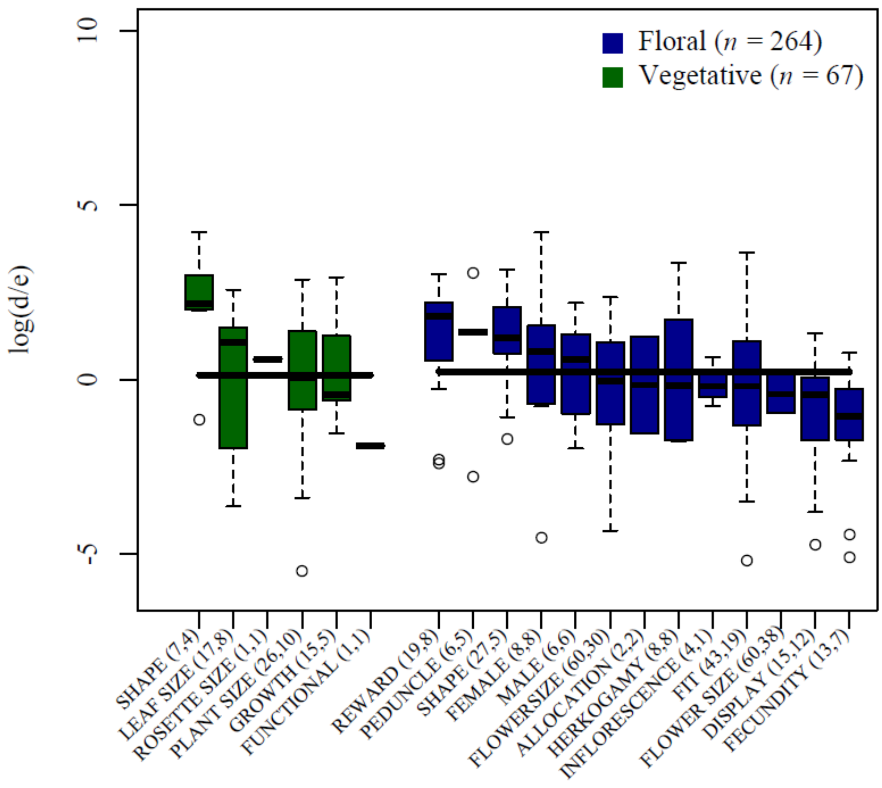


**Figure S9.** Ratios of proportional population divergence to evolvability for vegetative (green boxes) and floral (blue boxes) traits. The *y*-axis gives the natural log of proportional divergence over evolvability, where a value of 0 means that the two are of equal magnitude. Thick lines across boxes show the median of each trait category, and thick lines within boxes show median values for each trait subcategory. Boxes extend from the first to third quartile, range bars extend to 1.5 times the inter-quartile range, and data points outside this range are shown as open circles. Sample sizes are given in parentheses for each trait subcategory, with the first number giving the number of estimates, and the second number the number of unique studies.

*Comparing vegetative vs. floral traits for each mating-system category*

Patterns of divergence for floral vs. vegetative traits were similar across mating systems, with only a weak tendendy for the difference to be greater for the outcrossing species. Floral traits tended to have diverged more in selfers, while vegetative traits had diverged about equally in selfing, mixed-mating and outcrossing species.

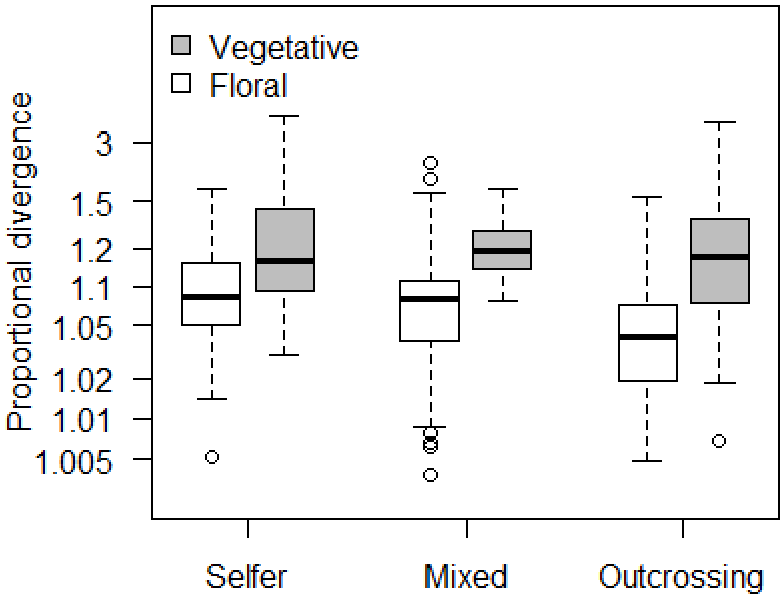


**Figure S10.** Proportional population divergence of vegetative and floral traits in selfing, mixed-mating, and outcrossing species. The *y*-axis gives the proportional divergence of the average population ($d_P$), where a value of 1.1 indicates that the trait mean of an average population has evolved to be c. 10% larger or smaller than the grand mean. Thick lines within boxes indicate median values for each trait subcategory. Boxes extend from the first to third quartile, range bars extend to 1.5 times the inter-quartile range, and data points outside this range are shown as open circles.

## Multivariate analyses

The following figures explore the results of the multivariate analyses for each trait functional group (and combination thereof), mating-system category, and study environment for the divergence data. For each of these, we plot the slope of the evolvability-divergence relationship (log-log regression slope of divergence on evolvability) for the G-directions (traits defined using the eigenvectors of the G-matrix) and the mean proportional evolvability along the divergence vector. These results correspond to figures 4 and 5 in the main text.

*Trait categories*

The slope of the evolvability-divergence relationships tended to be steeper in cases where floral and vegetative traits were combined ($n$ = 6). The mean proportional evolvability along the divergence vectors ($e[\Delta]/\bar{e}$) was somewhat greater in cases where only vegetative traits were considered ($n$ = 5).

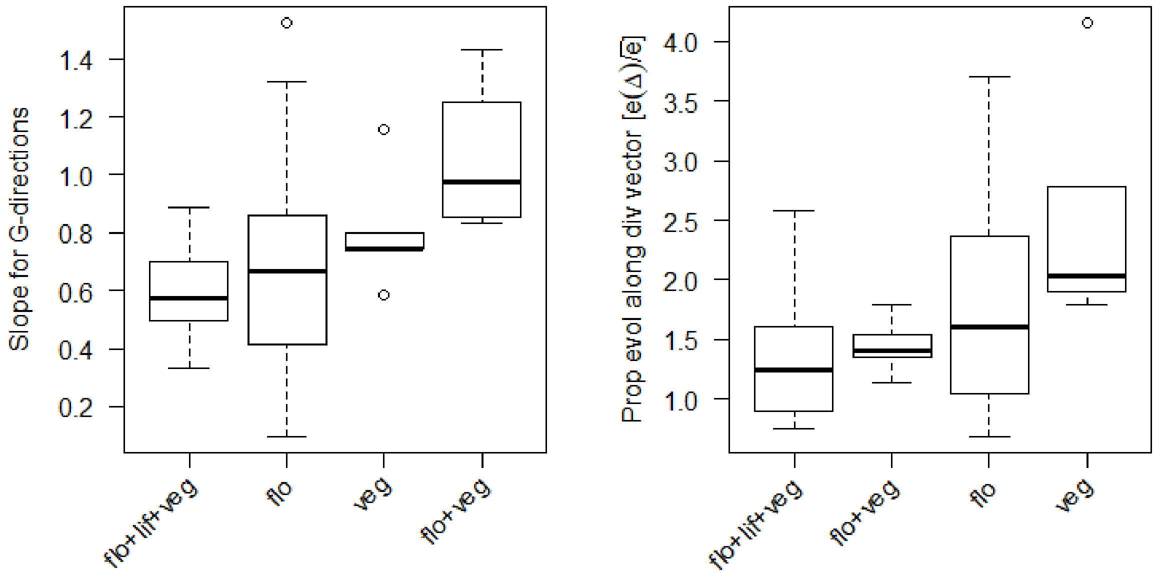


**Figure S11.** Evolvability-divergence patterns for studies including different combinations of trait types. Abbreviations: flo = floral traits, veg = vegetative traits, lif = life history traits. The left panel shows the slope of the evolvability-divergence relationship with traits defined along the eigenvectors of the G-matrix. The right panel shows the mean proportional evolvability along the divergence vector, i.e. the evolvability along the divergence vector divided by the mean evolvability of the focal-population G-matrix. Boxes extend from the first to third quartile, range bars extend to 1.5 times the inter-quartile range, and data points outside this range are shown as open circles.

*Mating systems*

The slope of the evolvability-divergence relationships tended to be steeper for mixed-mating and outcrossing species than for selfing species, and the slopes tended to be more variable for the outcrossers. The mean proportional evolvability along the divergence vectors ($e[\Delta]/\bar{e}$) was similar across mating systems.

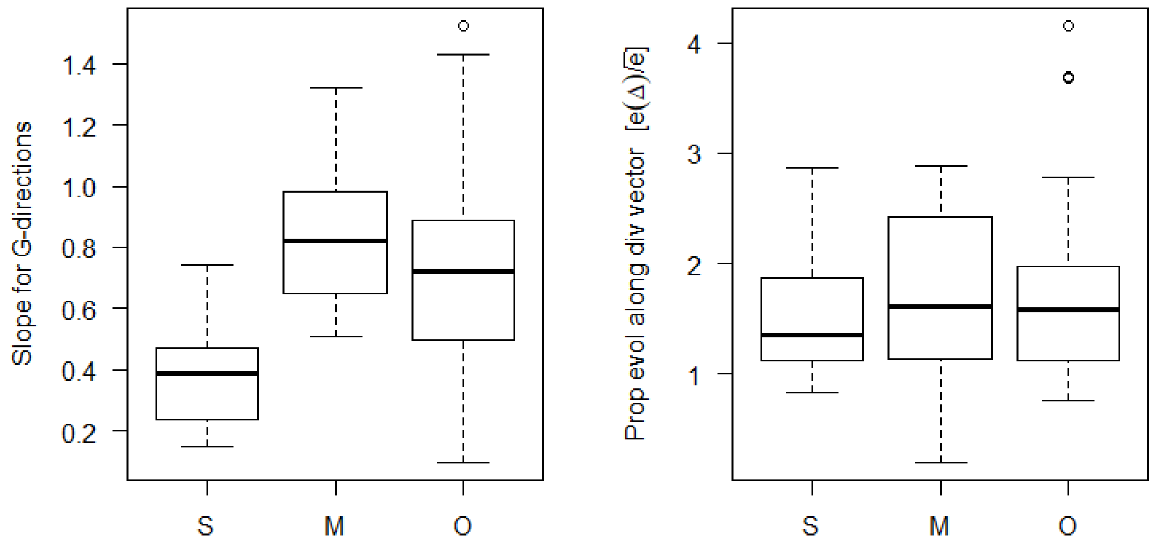


**Figure S12.** Evolvability-divergence patterns for selfing (S), mixed-mating (M) and outcrossing (O) species. The left panel shows the slope of the evolvability-divergence relationship with traits defined along the eigenvectors of the G-matrix. The right panel shows the mean proportional evolvability along the divergence vector, i.e. the evolvability along the divergence vector divided by the mean evolvability of the focal-population G-matrix. Boxes extend from the first to

third quartile, range bars extend to 1.5 times the inter-quartile range, and data points outside this range are shown as open circles.

*Study environment*

The slope of the evolvability-divergence relationships for the G-directions was similar when population divergence was studied in the field and in the greenhouse but was steeper for a single study where population divergence was studied in an outside common garden. The mean proportional evolvability along the divergence vectors ($e[\Delta]/\bar{e}$) was similar across study environments.

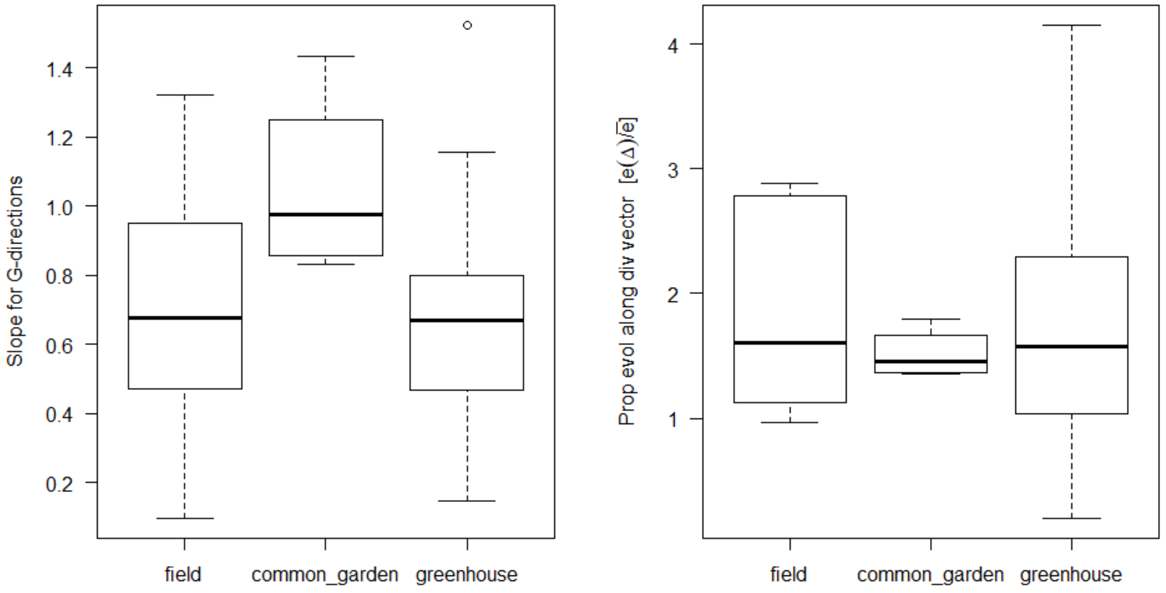


**Figure S13.** Evolvability-divergence patterns when the populations scored for divergence were studies in the field, in outside common gardens, and in the greenhouse. The left panel shows the slope of the evolvability-divergence relationship with traits defined along the eigenvectors of the G-matrix. The right panel shows the mean proportional evolvability along the divergence vector, i.e. the evolvability along the divergence vector divided by the mean evolvability of the focal-population G-matrix. Boxes extend from the first to third quartile, range bars extend to 1.5 times the inter-quartile range, and data points outside this range are shown as open circles.

## Appendix 5. Comparison of approaches and comparison to angle approaches

*Comparing the two multivariate approaches*

The slope of the evolvability-divergence relationships (log-log regression slope of divergence on evolvability) correlated positively with the average (over populations considered in a specific case study) proportional unconditional and conditional evolvabilities along the divergence vectors, suggesting that these parameters represent related aspects of evolvability-divergence relationships.

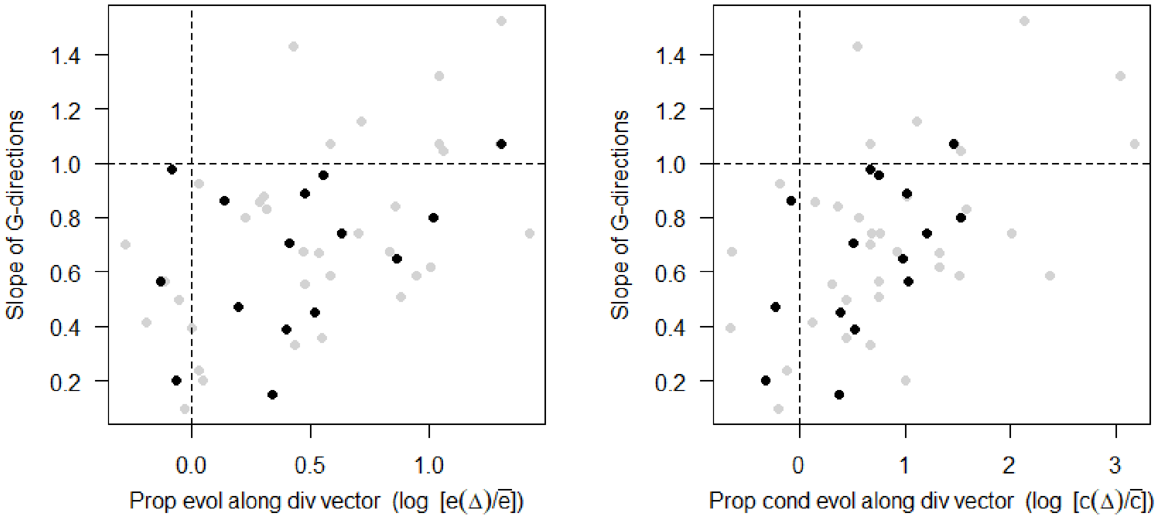


**Fig. S14.** Relationship, across divergence studies, between mean proportional evolvabilities along divergence vectors and the slope of the evolvability-divergence relationship for the G-directions. Grey symbols are individual cases (paired G- and D-matrices), black symbols are species means. Values greater than zero along the *x*-axis means that the evolvability along the divergence vector is greater than the mean of the focal-population G-matrix.

*Comparison to angle approaches*

Our multivariate approach to assessing evolvability-divergence relationships differs from more common approaches such as those based on comparing angles between the leading eigenvectors of **G** and **D** (1). Evolvability-divergence relationships and the angle between $\mathbf{g}_{max}$ and $\mathbf{d}_{max}$ represent different aspects of matrix similarity. While the relationships estimated as log-log slopes derive their theoretical relevance from established evolutionary theory (2, 3), comparison of angles remain a qualitative measure of alignment between matrices and, thus, provide only qualitative support for a constraint hypothesis. One problem with focusing on the angles between leading eigenvectors is that this ignores the higher geometry of multivariate divergence, and there may often be more than one direction in phenotype space of high evolvability (4).

Below, we visualize how the proportional evolvability along a divergence vector $\Delta\bar{\boldsymbol{x}}_{log}$ relates to the angle between the divergence vector and the leading eigenvector of the G-matrix ($\mathbf{g}_{max}$). When both the eigenvectors and $\Delta\bar{\boldsymbol{x}}_{log}$ are normalized to unit length, the angle between $\Delta\bar{\boldsymbol{x}}_{log}$ and the leading eigenvector is given by

$$\theta_{\Delta\bar{x}_{log}} = \frac{180}{\pi}\cos^{-1}(\boldsymbol{g}_{max}^{T}\Delta\bar{\boldsymbol{x}}_{log}).$$

We subtracted from 180° those angles >90° to obtain angles between 0° and 90°.

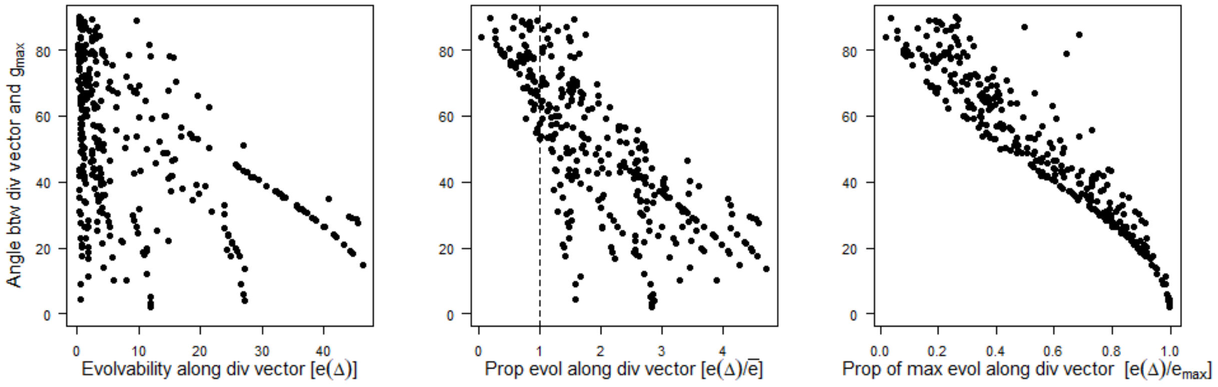


**Figure S15.** Comparison of the evolvability along a divergence vector and the angle between the divergence vector and the leading eigenvector of the focal-population G-matrix (**g**$_{max}$). The evolvability along the divergence vector is expressed as its raw value (left), in proportion of the mean evolvability of **G** (center), and in proportion of the maximum evolvability of **G** (right).

This exercise illustrates that a low angle between the divergence vector and **g**$_{max}$ is generally associated with greater-than-average evolvability along the divergence vector. However, greater-than-average evolvability can also occur with substantial angle, so that focusing only on the angle can lead to misleading conclusions.

Similarly, we visualize how the slope of the log-log regression of divergence on evolvability with traits defined along the eigenvectors of the G-matrix relates to the angle between the leading eigenvectors of the G- and D-matrices. We computed the angle as

$$\theta_{GD} = \frac{180}{\pi}\cos^{-1}({\boldsymbol{g}_{max}}^{T}\boldsymbol{d}_{max}).$$

This exercise illustrates that the angle between matrices is numerically related to the measures discussed here, so that as the angle tends toward zero, the evolvability along the divergence vectors will tend toward **g**$_{max}$, the overall $r^2$ of the evolvability-divergence relationship will tend toward 1, and, less intuitively, the slopes become steeper (apparently converging at or a little above 1).

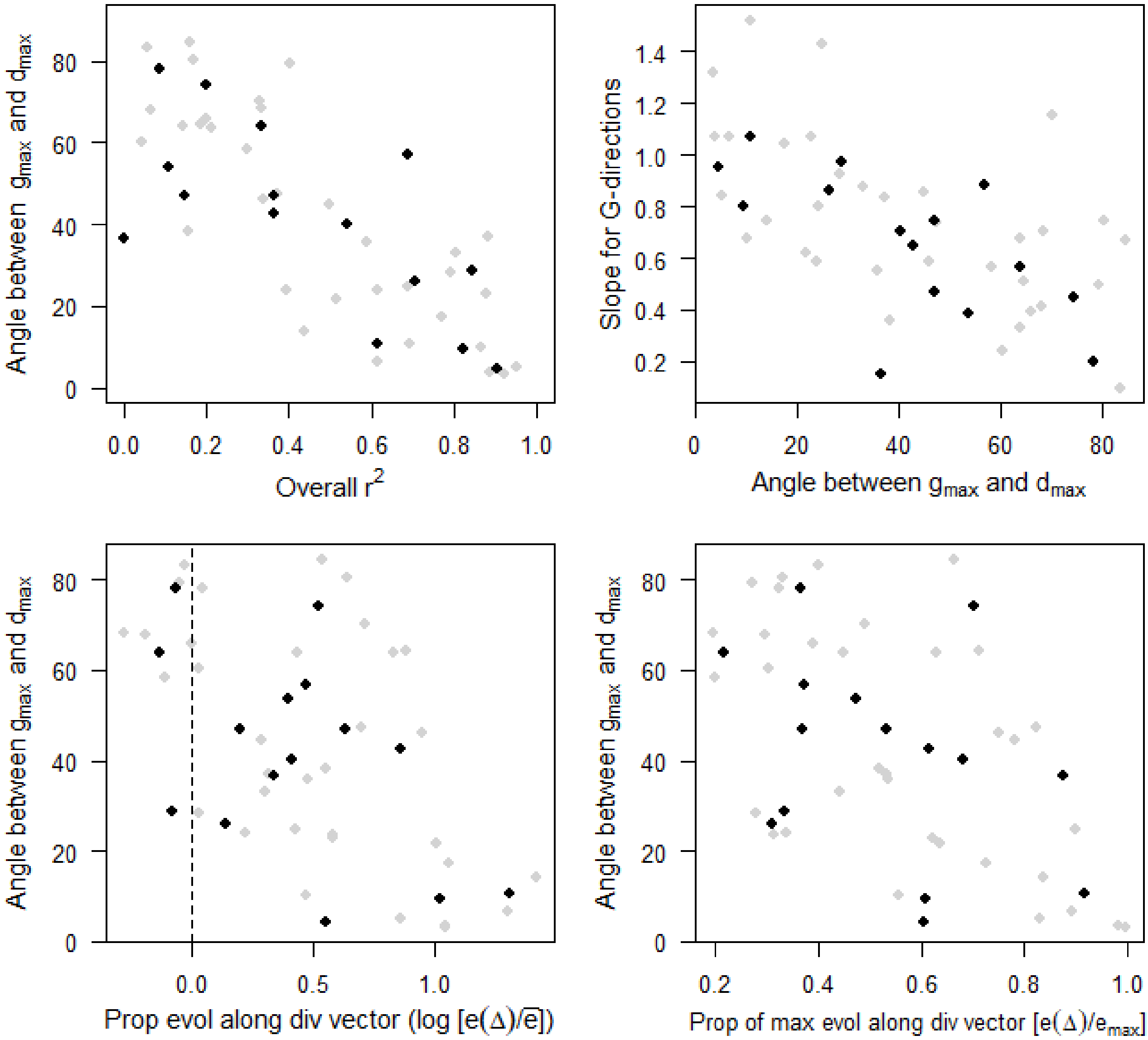


**Figure S16.** Comparison of the angle between the leading eigenvectors of the G- and D-matrices, the evolvability-divergence relationship estimated for the G-directions, and the proportional evolvability along a divergence vector. Grey symbols are individual cases (paired G- and D-matrices), black symbols are species means. On the upper left panel, the 'Overall $r^2$' is for the regression of divergence on evolvability. On the lower left panel, values greater than zero along the x-axis means that the evolvability along the divergence vector is greater than the mean of the focal-population G-matrix.

## Appendix 6. Extended analytical methods

*General approach to estimating G- and D-matrices from original data*

We estimated G-matrices from raw data by fitting multivariate ‘animal models’ with the `MCMCglmm` R package (5). We ran the models for an increasing number of Monte Carlo Markov Chain iterations (by increasing the thinning interval between each of 1000 posterior samples) until visual inspection of the posterior trace plots indicated convergence and effective sample sizes close to the expected 1000 indicated adequate mixing. To estimate D-matrices from original data, we fitted similar models with a random term for population. As priors for the Bayesian mixed models (`MCMCglmm`), we used the default for the fixed effects, which is zero-mean normal distributions with very large variances, scaled non-central F-distributions for the variance components, and inverse-Wishart distributions with parameters $\mathbf{\Psi}$ and $v$ for the residuals. In R-syntax the prior density of variance components (v) was given by `df(v/alpha.V, df1 = 1, df2 = n, ncp = (alpha.mu^2)/alpha.V)`, where `alpha.V` is the scaling parameter, `df1` and `df2` are the degrees of freedom, and `ncp` is the non-centrality parameter. The value of `alpha.V` was set to 400, `n` was the number of traits and `alpha.mu` was set to zero. For the inverse-Wishart distributions the matrix parameter $\mathbf{\Psi}$ was set as the identity matrix with dimensions equal to the number of traits, and the value of $v$ was set to $n$ - 0.998, where $n$ is number of traits in the analysis. This prior is expected to have good properties for estimating small variances, though constrains the estimated variance-covariance matrix to be positive definite (symmetric and with all eigenvalues positive). Because our analyses concerns the relationship between variances within and among populations (whether traits or dimensions with low variance within population also has diverged to a lesser extent), we did not perform hypothesis testing for the existence of genetic variance in particular traits.

*Error-corrected D-matrices from summary statistics*

When original data was not available (Appendix 1), we obtained error-corrected D-matrices by fitting multivariate mixed models on the form $z_{ij} \sim d_{ij} + m_{ij}$, where the subscripts $i$ and $j$ denote trait and population respectively, $z$ is the trait measurements (observed population means), $d$ is the error-corrected trait means of each population and $m$ is the measurement error. The $d$ and $m$ are random effects distributed as $d \sim N(\mu, \mathbf{D})$ and $m \sim N(0, \mathbf{P}/n)$.

*Dimension reduction*

A few of the published G-matrices were rank deficient, i.e. they had one or more negative eigenvalues. We reduced the dimensionality of these matrices to only those dimensions associated with positive eigenvalues. To do so, we rotated both matrices (**G** and **D**) onto the same set of eigenvectors, and dropped rows and columns associated with negative eigenvalues. In the analysis of divergence vectors, we similarly projected the divergence vector onto the eigenvectors of the G-matrix and dropped the directions associated with negative eigenvalues.

# SI References


1. D. Schluter, Adaptive radiation along genetic lines of least resistance. *Evolution* **50**, 1766-1774 (1996).
2. G. H. Bolstad *et al.*, Genetic constraints predict evolutionary divergence in *Dalechampia* blossoms. *Philos Trans R Soc Lond B Biol Sci* **369**, 20130255 (2014).
3. D. Houle, G. H. Bolstad, K. van der Linde, T. F. Hansen, Mutation predicts 40 million years of fly wing evolution. *Nature* **548**, 447-450 (2017).

4. T. F. Hansen, K. L. Voje, Deviation from the line of least resistance does not exclude genetic constraints: a comment on Berner *et al.* (2010). *Evolution* **65**, 1821-1822 (2011).
5. J. D. Hadfield, MCMC methods for multi-response generalized linear mixed models: The MCMCglmm R package. *Journal of Statistical Software* **33**, 1-22 (2010).
